# Detailed lipid investigation of edible seaweeds by photochemical derivatization and untargeted lipidomics

**DOI:** 10.1007/s00216-024-05573-6

**Published:** 2024-10-11

**Authors:** Carmela Maria Montone, Chiara Cavaliere, Andrea Cerrato, Aldo Laganà, Susy Piovesana, Enrico Taglioni, Anna Laura Capriotti

**Affiliations:** Department of Chemistry, Sapienza University of Rome, Università Di Roma “La Sapienza”, Piazzale Aldo Moro 5, 00185 Rome, Italy

**Keywords:** Aza-Paternò–Büchi, Fatty acids, High-resolution mass spectrometry, Carbon–carbon double bonds, Data processing

## Abstract

Seaweeds are macrophytic algae that have been gaining interest as alternative healthy foods, renewable drug sources, and climate change mitigation agents. In terms of their nutritional value, seaweeds are renowned for their high content of biologically active polyunsaturated fatty acids. However, little is known about the regiochemistry—the geometry and position of carbon–carbon double bonds—of free and conjugated fatty acids in seaweeds. In the present work, a detailed characterization of the seaweed lipidome was achieved based on untargeted HRMS-based analysis and lipid derivatization with a photochemical aza-Paternò–Büchi reaction. A triple-data processing strategy was carried out to achieve high structural detail on the seaweed lipidome, i.e., (i) a first data processing workflow with all samples for aligning peak and statistical analysis that led to the definition of lipid sum compositions (e.g., phosphatidylglycerol (PG) 34:1), (ii) a second data processing workflow in which the samples of each seaweed were processed separately to annotate molecular lipids with known fatty acyl isomerism (e.g., PG 16:0_18:1), and (iii) the annotation of lipid regioisomers following MS/MS annotation of the lipid derivatives obtained following the aza-Paternò–Büchi reaction (e.g., PG 16:0_18:1 ω-9). Once the platform was set up, the lipid extracts from 8 seaweed species from different seaweed families were characterized, describing over 900 different lipid species, and information on the regiochemistry of carbon–carbon double bonds uncovered unknown peculiarities of seaweeds belonging to different families. The overall analytical approach helped to fill a gap in the knowledge of the nutritional composition of seaweeds.

**Graphical Abstract:**

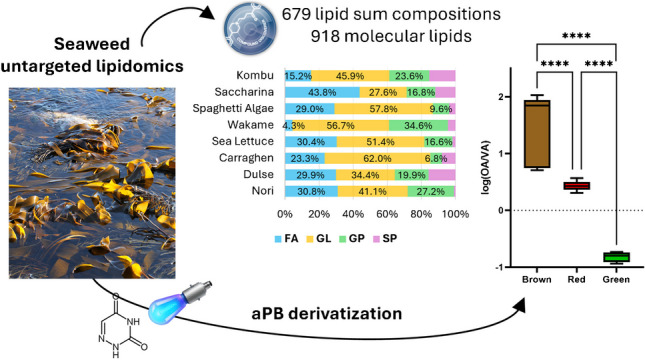

**Supplementary Information:**

The online version contains supplementary material available at 10.1007/s00216-024-05573-6.

## Introduction

Seaweeds are macrophytic algae lacking true roots, stems, and leaves that belong to three main evolutionarily diverse groups, i.e., Ochrophyta (brown algae), Chlorophyta (green algae), and Rhodophyta (red algae), the latter comprising the families of Floridophyceae and Bangiophyceae [1]. According to AlgaeBase (https://www.algaebase.org/), Rhodophyta and Chlorophyta are included in the kingdom Plantae, while Ochrophyta are in the kingdom Chromista, even though the three groups are related through the endosymbiotic events that gave rise to plastids [2]. Seaweeds are attracting increasing attention as alternative healthy food, renewable drug sources, and climate change mitigation agents that provide essential ecosystem services [3]. In this context, seaweeds serve as marine resources with the potential to advance the goals of both the Sustainable Blue Economy and the Bio-Based Circular Economy. At present, approximately 221 seaweed species possess commercial value, contributing to a global seaweed industry valued at over USD 6 billion annually. Notably, more than 150 of these species (representing 85%) are utilized as food products for human consumption [4]. Edible seaweeds can be used for food consumption and as a dietary supplement, providing a variety of nutrients essential to human health, such as natural vitamins [5], minerals [6], polysaccharides [7], pigments [8], polyphenols [9], proteins, and peptides [10] which have been proven to possess various beneficial biological [11]. Among the various compounds in seaweed biomass, lipids have received less attention than other molecules mainly due to their relatively low abundance, typically between 1 and 10% of dry weight, depending on the species [12]. Although the overall lipid content in algae might be relatively low compared to some other organisms, seaweeds have been attracting a lot of attention since they represent an innovative feedstock for the food market as a fatty acid (FA) source [13]. Seaweed lipids serve as a notable source of omega-3 (ω-3) and omega-6 (ω-6) polyunsaturated fatty acids (PUFAs), such as α-linolenic acid (ALA; 18:3 ω-3) and linoleic acid (LA; 18:2 ω-6), both of which are essential FAs that must be obtained through dietary intake as mammals cannot synthesize them. Additionally, macroalgae provide long-chain ω-3 and ω-6 PUFAs, including arachidonic acid (AA; 20:4 n-6) and eicosapentaenoic acid (EPA; 20:5 ω-3), which play an essential role in the prevention and management of non-communicable diseases [14, 15].

Recently, the polar lipidome of different algal species was also comprehensively characterized, highlighting the presence of glycerophospholipids (GPs), glycerolipids (GLs), and sphingolipids (SPs) with a different composition that is unique to each macroalgal species depending of varying abiotic conditions [16–19]. However, these studies, which were carried out using the standard high-resolution mass spectrometry (HRMS)-based lipidomics approach, did not consider the regiochemistry of FAs and double bonds in FAs in their free form and bound to GPs, GLs, and SPs, i.e., the geometry and position of carbon–carbon double bonds.

Understanding the distribution of regioisomers and the position of double bonds in seaweed lipids is critical for evaluating their nutritional quality, dietary intake, and potential biological effects [20, 21]. For example, double bond position in seaweed GPs and GLs can significantly affect membrane fluidity, permeability, and stability, ultimately influencing various cellular processes and functions [22]. Various methodologies have been introduced over the years for identifying carbon–carbon double bonds, such as ozone-induced fragmentation [23], ultraviolet photodissociation [24], and Paternò–Büchi (PB) derivatization [25, 26] and its modifications [27]. While most studies for pinpointing carbon–carbon double bonds in unsaturated lipids were applied to clinical samples [28, 29], only a few papers have dealt with the double bond location of FAs in lipid structures in plant samples. Coniglio et al. [30] carried out a positional assignment of carbon–carbon double bonds in fatty acyl chains of arsenosugar phospholipids in seaweed extracts by epoxidation reactions; Jeck and her co-workers [31, 32] applied a postcolumn Paternò–Büchi derivatization to pinpoint the double bond positions in diacylglyceryltrimethylhomoserine (DGTS) and phosphatidylglycerol (PG) species in green microalgae *Chlamydomonas reinhardtii*. Recently, our research team applied a one-phase extraction coupled with a photochemical reaction for pinpointing carbon–carbon double bonds in hempseed [33].

In this study, an extensive and comprehensive untargeted lipidomics investigation was conducted on lipid extracts derived from seaweeds belonging to eight distinct species from the three main seaweed families (Ochrophyta, Chlorophyta, and Rhodophyta). Following lipid extraction, lipids were analyzed by HRMS in their native form and following aza-Paternò–Büchi (aPB) derivatization reaction using 6-azauracil (6-AU). Particular attention was paid to the data processing workflow for lipid annotation. As such, data processing represents a crucial step in lipidomics based on the need for reducing sample complexity and dealing with lipid annotation [34]. A triple-data processing strategy was carried out to achieve high structural detail on seaweed lipidome to annotate lipid sum compositions, molecular lipids, and lipid regioisomers, respectively. This holistic analytical approach effectively addressed gaps in understanding the composition of the polar lipidome in seaweeds and its variation across different strains as well as highlighting the need for proper data processing when large datasets of highly different samples are simultaneously analyzed.

## Materials and methods

### Lipid nomenclature

The shorthand notation of the lipid species followed the LIPID MAPS guidelines [35]. The location of carbon–carbon double bonds was indicated using the ω-nomenclature, where carbon atoms are counted from the methyl end of the fatty acyl chain. Underscore (“_”) indicates unspecified *sn*-positions. Lipid structural assignments followed the hierarchical level nomenclature proposed by Rustam and Reid [36]. Lipids whose fatty acyl composition was unspecified were referred to as sum compositions, whereas those with known fatty acyl composition were referred to as molecular lipids. These two identification levels correspond to the “species level” and “molecular species level” reported by the LIPID MAPS classification system [37].

### Chemicals and materials

Optima mass spectrometry (MS) grade water, methanol (MeOH), and isopropanol (iPrOH) were purchased from Thermo Fisher Scientific (Waltham, MA, USA). Glacial acetic acid, ammonium acetate, 6-AU, and chloroform were purchased from Merck. A mixture of isotope-labeled lipids (Splash Lipidomix) (internal standard, IS) was purchased from Merck (Darmstadt, Germany). GHP filters (0.20 µm, Ø 13 mm) were acquired from Acrodisc, Pall Laboratory. Seaweeds were purchased from ConsonniBioalghe Srls (Milan, Italy). Four Ochrophyta brown algae of the class Phaeophyceae were analyzed, i.e., *Laminaria digitata* (kombu), *Saccharina latissima* (saccharina), *Himanthalia elongata* (spaghetti algae), and *Undaria pinnatifida* (wakame), as well as one Chlorophyta green algae of the family Chlorophyceae, i.e., *Ulva lactuca* (sea lettuce), and three Rhodophyta red algae of the families Floridophyceae, i.e., *Chondrus crispus* (carraghen) and *Palmaria palmata* (dulse), and Bangiophyceae, i.e., *Porphyra umbilicalis* (nori).

### Seaweed lipid extraction

For each seaweed, 500 mg aliquots (*n* = 6) were pooled, ground, homogenized, and extracted using a modified biphasic extraction method outlined by Bligh and Dyer (B&D) [38]. Briefly, 500 mg of each sample was added to 3.45 ml of MeOH and vortexed for 2 min at room temperature (RT). Then, 3.45 ml of CHCl_3_ was added, and the mixture was vortexed for 2 more min at RT. Finally, 3.1 ml of water was added into the glass tube, and the mixture was vortexed for 20 min. Samples were centrifuged at 3000 × *g* at 20 °C for 15 min, allowing phase separation. The lower layer was finally transferred to a new glass tube and evaporated with a SpeedVac SC 250 Express (Thermo 164 Avant, Holbrook, NY, USA). Four experimental replicates were then resuspended in 400 µl of MeOH/H_2_O/CHCl_3_ (80:15:5, *v*/*v*/*v*) mixture and filtered through GHP filters (0.20 µm, Ø 13 mm). Then, a 195 µl aliquot was put in a glass vial, and 5 µl of IS solution was added to each sample. The IS solution was needed for quality control monitoring during and after the analyses following the guidelines of the Metabolomics Quality Assurance and Quality Control Consortium [39]. The other two experimental replicates were subject to aPB derivatization as described in the following section.

### Offline aza-Paternò–Büchi derivatization

aPB reaction with 6-AU was conducted on lipid extract as previously described [27]. The reaction solution containing aPB reagent (6-AU, 24 mM) was prepared in MeOH. After evaporation, seaweed lipid extracts were resuspended in 950 µl consisting of 710 µl of MeOH, 180 µl of water, and 60 µl of CHCl_3_. Subsequently, each sample solution was filtered directly into a quartz cuvette through GHP filters (0.20 µm, Ø 13 mm). Later, 250 µl of 6-AU solution (24 mM in MeOH) was mixed with each extract to reach a phase composition at MeOH/H_2_O/CHCl_3_ (80:15:5, *v*/*v*/*v*) and purged with nitrogen gas to remove residual oxygen. The cuvette was exposed to 254 nm radiation using a Spectroline E-Series UV lamp emitting shortwave UV (Thermo Fisher Scientific) for 30 min at room temperature. Subsequently, the reaction mixtures were collected, and a 195 µl aliquot was transferred into a glass vial containing 5 µl of an IS mixture before ultra-high-performance liquid chromatography (UHPLC)-HRMS analysis.

### UHPLC-HRMS analysis

Lipid separation was carried out by a Vanquish Binary Pump H (Thermo Fisher Scientific, Bremen, Germany), equipped with a thermostated autosampler and column compartment, on a C8 Hypersil GOLD (100 × 2.1 mm, 1.9 µm particle size; Thermo Fisher Scientific) at 50 °C with a flow rate of 300 µl min^−1^. The mobile phases consisted of H_2_O/CH_3_COOH (99.85:0.15, *v*/*v*) with 5 mmol l^−1^ CH_3_COONH_4_ (phase A) and MeOH/i-PrOH/CH_3_COOH (79.85:20.00:0.15, *v*/*v*/*v*) with 5 mmol l^−1^ CH_3_COONH_4_ (phase B). The chromatographic gradient was as follows: 1 min at 40% phase B, increasing to 70% phase B over 4 min, then to 99% phase B over 18 min; maintaining 99% phase B for 10 min (washing step); reducing to 40% phase B over 1 min; and holding at 40% phase B for 8 min (equilibration step). The injection volume was 10 µl. The UHPLC system was coupled to the Q Exactive hybrid quadrupole-Orbitrap mass spectrometer (Thermo Fisher Scientific) with the following source settings in negative ion mode (electrospray ionization (ESI) −): spray voltage, 2.5 kV; capillary temperature, 320 °C; sheath gas flow rate, 35 arbitrary units (a.u.); auxiliary gas flow rate, 25 a.u.; and auxiliary gas heater temperature, 400 °C. Full-scan MS data were acquired in the 200 − 1200 m/z range with a resolution (full width at half maximum, FWHM) of 35,000, automatic gain control (AGC) target value at 500,000, the maximum ion injection time at 200 ms, and the isolation window width at 2 m/z. Top 5 data-dependent acquisition (DDA) MS/MS fragmentation was achieved with a resolution (FWHM) of 17,500, AGC target value at 100,000, and dynamic exclusion at 2 s. Collision energy fragmentation was achieved in the higher-energy collisional dissociation (HCD) cell at 30 NCE. A process blank sample, obtained after a solvent sample was subject to the whole analytical platform, was analyzed together with the samples. Raw data files were acquired by Xcalibur software (version 3.1, Thermo Fisher Scientific).

### Lipid identification

Raw data obtained from three consecutive injections and the process blank sample were preprocessed by Compound Discoverer (v. 3.1; Thermo, Waltham, USA) using a customized data processing workflow dedicated explicitly to the tentative identification of fatty acids and polar lipids. Indeed, a mass list concerning FAs, GPs, SLs, and GLs was built in Excel based on the LIPIDS MAPS database [40] by combining one or two among 28 saturated FA (SFAs), monounsaturated FA (MUFA), and PUFA with the polar heads corresponding to six GP classes (phosphatidic acid (PA), phosphatidylcholine (PC), phosphatidylethanolamine (PE), PG, phosphatidylinositol (PI), and PS) and five sphingolipid (SL) classes (ceramides, dihydroceramides, phytoceramides, sphingomyelins, and ceramide phosphoinositols). For PE, the N-acetylation of the free amino group was also considered. Moreover, six classes of glycolipids (GLs) were included, i.e., monogalactosylmonoacylglycerols (MGMGs), monogalactosyldiacylglycerols (MGDGs), digalactosylmonoacylglycerols (DGMGs), digalactosyldiacylglycerol (DGDG), sulfoquinovosylmonoacylglycerols (SQMGs), and sulfoquinovosyldiacylglycerol (SQDG). Feature alignment was obtained by the adaptive curve regression model; whenever the adaptive curve model failed, the linear model was automatically selected instead. After spectral selection and alignment, employing the tools “Fill Gaps” and “Mark Background Compounds,” adducts were detected and grouped, and the features were then filtered to remove those whose areas in the process blank were greater than 10% of the average peak areas in the samples present in the process blank. Moreover, Compound Discoverer allowed the prediction of elemental compositions and the match of the extracted masses and elemental compositions to those present in the lipid mass list. To further facilitate the manual annotation of lipids by reducing the number of features, every feature whose mass was not present in the lipid database was filtered out. Due to their zwitterionic structure, PCs inhibit their deprotonation when subject to ESI and in-source fragmentation of the ammonium group or its interaction with negatively charged ions deriving from buffer modifiers (acetate or formate) [41]. Indeed, PCs undergo an unusual ionization pathway that leads to incorrect annotation in the preprocessing step of data analysis (e.g., when the software program annotates the adducts and molecular formulas). To overcome this matter, PCs were included in the database by subtracting 14.0156 from their exact masses. By doing this, the demethylated ions that the software would incorrectly annotate as deprotonated ions were associated with the correct structure, and the actual molecular weights and formulas were manually corrected after the spectral annotation. The same rationale was applied to SM, which also possesses a phosphocholine group. Lastly, filtered features resulting from the data processing step were putatively identified and annotated by matching the experimental tandem mass spectra with open-access mass spectral databases and/or based on known lipid fragmentation patterns [42–44]. A second data processing step was performed for each group of sample experimental and instrumental replicates to annotated molecular lipids of diacyl lipid sum compositions (GP and GL). For this purpose, a reduced lipid mass list that included the ID, molecular formulas, and molecular weight of the 543 annotated GL and GP sum compositions was employed as a mass list in place of the complete lipid database. Finally, to determine carbon − carbon double bonds in fatty acyl chains, aPB reaction products (corresponding to a relative mass shift of + 113.0225) were manually searched in the derivatized sample MS data. Diagnostic product ions and relative abundances of the annotated lipid isomers were evaluated based on previous results [27].

### Statistical analysis

MetaboAnalyst 6.0 was employed for statistical analysis and data visualization [45]. Following the specific indications furnished by the developers, the data matrix was submitted as a text file. The interquartile range (IQR) was selected for data filtering, whereas the autoscaling algorithm was selected for data scaling. The data matrices obtained after the annotation of the underivatized lipids were submitted to MetaboAnalyst to obtain hierarchical clustering information (dendrogram and heatmap), principal component analysis (PCA), as well as correlation heatmaps. Box and whiskers plots with *t*-test analysis were obtained by GraphPad Prism 10 (GraphPad Software, La Jolla, CA, USA).

## Results and discussion

### Annotation of lipid sum compositions from seaweed lipid extract

Understanding and characterizing the categories of lipids in seaweeds is essential for elucidating their ecological significance, exploring their potential applications in the biofuel field, and highlighting their nutritional value. Seaweed lipids were extracted using the B&D protocol [16]. Preliminary results, in fact, showed that B&D performed significantly better than monophasic protocols (Supplementary Fig. 1), possibly due to the need for highly nonpolar solvents, e.g., CHCl_3_, to effectively break down the cell walls of seaweed cells. These results marked a great contrast with our previous study on hempseeds [33], in which one-phase extraction with methanol resulted in much more effective extraction of free FA and those conjugated to GP, and confirmed that the characteristics of the matrix greatly affected the efficiency of extraction procedures. For the structural characterization of seaweed polar lipidome, untargeted liquid chromatography (LC)-HRMS analysis followed by data processing by Compound Discoverer software was employed on underivatized extracts using a customized method specifically dedicated to lipid analysis. The Compound Discoverer software allows small molecule identification based on exact masses and fragmentation spectra. It is based on a system of blocks and nodes that can be customized by the user for the development of specific data-processing methods. Data preprocessing allowed feature alignment, background removal, and adduct grouping. Moreover, Compound Discoverer enables the prediction of elemental compositions and the match of the extracted masses and elemental compositions to those present in a homemade lipid mass list that was compiled ex novo based on the LIPID MAPS lipid classification [40]. For this purpose, an extensive customized database based on the knowledge of the algal lipid composition [46, 47] was built for six GP classes, five SP classes, and six GL classes. Due to the chosen method of analysis (ESI −), betaine lipids (BLs), which are solely ionized in positive ion mode, were not included in the present study. The lipid database comprising the associated molecular formulas and exact masses of each lipid was then uploaded to Compound Discoverer as a mass list. The “Search Mass List” tool filters the extracted and aligned features to remove calculated masses not included in the database. Finally, filtered features corresponding to FAs and GPs were manually annotated by investigating the experimental MS/MS spectra based on their well-known fragmentation pathways [42–44].

Compound Discoverer allowed simultaneous processing of all underivatized lipid extract datasets (two instrumental replicates of each of the four experimental replicates per seaweed species). The data processing workflow, therefore, aligned all peaks from the different runs into a single feature and associated each feature to the most intense MS/MS spectra in the datasets, thus enabling a much faster manual annotation. Most lipids with isomeric fatty acyl chain compositions, i.e., PG 14:0_18:1 and PG 16:0_16:1 at RT 17.0, were not separated by the C8 column and eluted as a single peak for most GPs and GLs [46], whereas SP isomers were usually separated, i.e., Cer d18:0/24:1 and Cer d18:1/24:0 at RT 21.2 and 21.6, respectively. The co-elution of the *sn*-isomers of GP and GL and the fact that Compound Discoverer selected the most intense MS/MS in the whole datasets meant that, at this stage, it was not possible to distinguish fatty acyl isomers of GP and GL, taking into account that different algae could present different fatty acyl chain compositions of the same eluting lipid peaks. Therefore, the data processing workflow of all seaweed lipid extract would allow the annotation of lipid sum compositions, i.e., molecular lipids with the FA chains reported as a sum. Each peak corresponded to a single sum composition, such as PG 32:1 for the pair of co-eluting isomers PG 14:0_18:1 and PG 16:0_16:1. A total of 679 lipid sum compositions (lipid peaks) were annotated in the 8 analyzed macroalgae, including 34 FA, 331 GP, 102 SL, and 212 GL. Supplementary Tables 1–4 list the annotated lipid sum compositions alongside their annotation data, including RT, molecular formula, adduct, molecular weight, experimental m/z, and diagnostic product ions. Among the GP classes, PGs were the most numerous with 94 annotated sum compositions, followed by PEs with 71 annotated lipids including four N-acyl PE (NAPE). PC, PI, and PA shared a similar number of identifications, with 60, 54, and 52 annotated sum compositions, respectively. Four main classes of SL were annotated including their α-hydroxylated counterparts, i.e., 39 ceramides, 30 dihydroceramides, 23 ceramide phosphoinositols, and 10 phytoceramides, whereas no sphingomyelins were annotated. Finally, sulfolipids were the most numerous GL with 67 SQDG and 32 SQMG annotations, followed by DGDG (57), DGMG (22), MGDG (20), and MGMG (14).

### Lipid category and class composition of the seaweed extracts

**Fig. 1:**
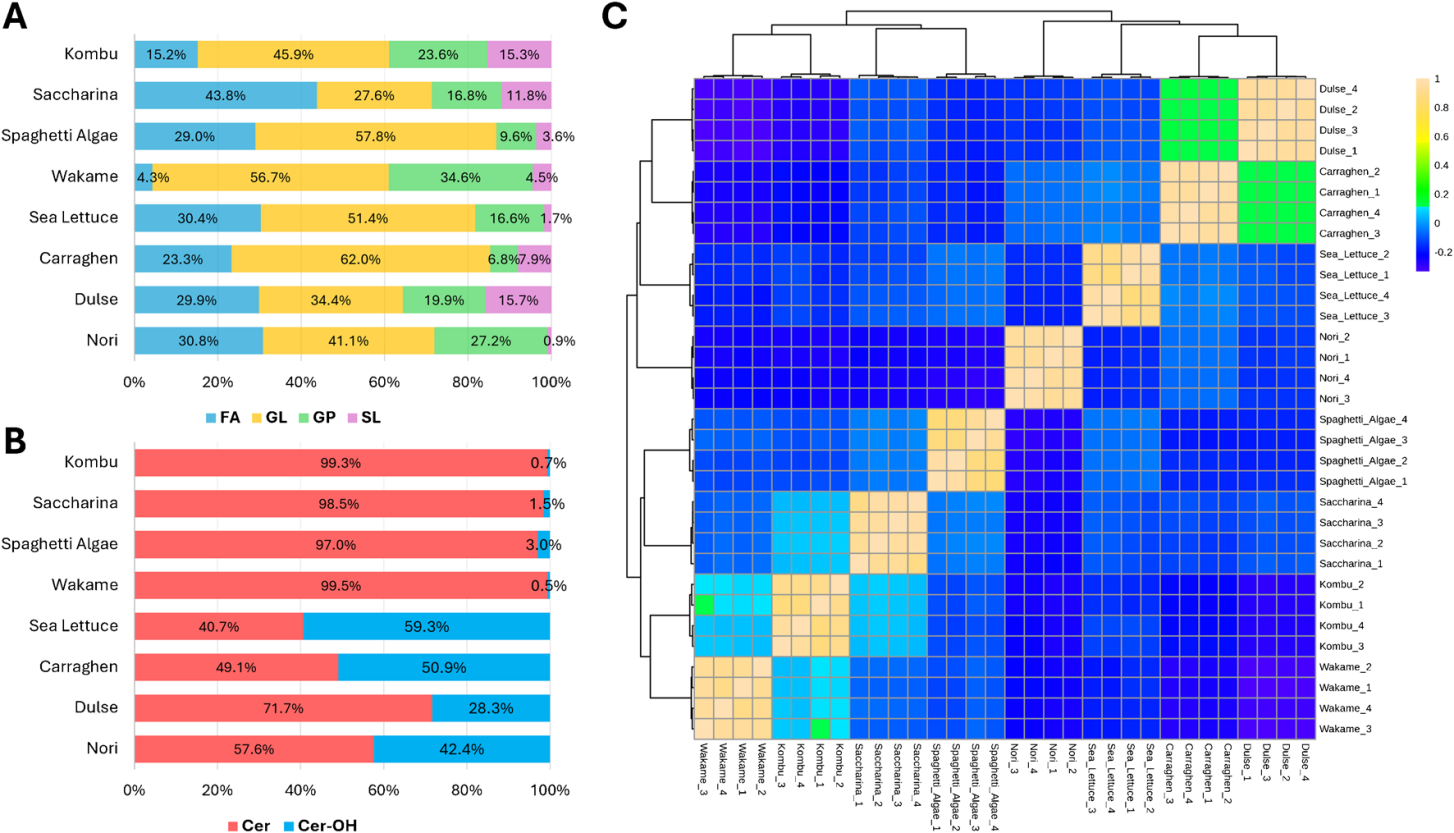
**A** Stacked bar charts displaying the relative peak areas of the four annotated lipid categories (FA, GL, GP, SP) and **B** the relative peak areas of hydroxylated and non-hydroxylated ceramides. **C** Correlation heatmap with hierarchical clustering dendrogram using the 679 annotated lipid data matrices

By taking into consideration the lipid category peak areas in the 8 analyzed algae, GLs were generally the most abundant class in all but one sample (saccharina) with a relative total peak area between 27.6 and 62.0%, followed by FA (4.3–43.8%), GP (6.8–34.6%), and SL (1.7–15.7%), as shown in Fig. 1A. To investigate the role of the annotated lipids in clustering the analyzed algae, a data matrix with the peak area of the 677 annotated lipids in the 32 analyzed samples (4 replicates of each of the 8 analyzed macroalgae) was submitted to MetaboAnalyst 6.0. The PCA of the whole lipid datasets did not show a clear trend (Supplementary Fig. 2), with nori standing out from the other 7 algae alongside principal component 1 (PC1; 26.1% of the total variance). On the other hand, partial clustering can be observed alongside principal component 2 (PC2; 20.3% of the total variance), with brown algae (wakame, kombu, spaghetti algae, and saccharina) showing positive PC2 values or values around zero, while green and red algae (sea lettuce, dulse, and carraghen) had negative values. However, if a correlation heatmap is built (Fig. 1C), the correlation among the analyzed samples is rather scarce, and only red algae dulse and carraghen had a Pearson correlation coefficient (PCC) higher than 0.2. Therefore, despite the hierarchical clustering dendrogram discriminating brown algae from red and green algae, the whole lipidome appeared unsuitable for clustering algae from different classes. In terms of the GP classes, PGs were the most abundant in terms of the total peak area for all analyzed samples, whereas the areas of the other classes varied significantly between the analyzed algae (Supplementary Fig. 3). As such, a reduced data matrix consisting of the sole 331 GP furnished even poorer results in terms of clustering of the samples and their correlation (Supplementary Fig. 4). Among the GL classes, sulfolipids (SQDG + SQMG) were by far the most abundant lipid class (Supplementary Fig. 5A), in line with previous findings [46, 47]. The evaluation of the ratio between MGDG and DGDG furnished the first valuable results for clustering the analyzed algae, with brown algae having comparable abundances of the two classes whereas red and green algae with an approximate 4:1 ratio (DGDG/MGMG; Supplementary Fig. 5B).

By comparing the results obtained by the PCA and hierarchical clustering statistical analysis of the reduced data matrix with all GL and further reduced data matrix that excluded sulfolipids, scarce differences were observed on the PCA score plots (Supplementary Fig. 6A vs Supplementary Fig. 7A) whereas the hierarchical clustering analysis showed different results. In particular, the dendrogram that resulted from the whole GL dataset showed a distinction between brown and green/red algae (Supplementary Fig. 6B), the former being somehow correlated (PCC around 0.2) as well as dulse and carraghen. On the other hand, the reduced data matrix of the sole galactosyl lipids furnished three clusters (Supplementary Fig. 7B), with the first cluster (wakame, kombu, and spaghetti algae) being somehow correlated with the third one (saccharina and nori). SP classes exhibited interesting trends among the analyzed samples. As such, non-hydroxylated SP classes, i.e., Cer [NS], Cer [NDS], and Cer-PI, constituted over 97% of the total SP peak area in brown algae, whereas hydroxylated classes, i.e., Cer [NP], Cer [AS], Cer [ADS], Cer [AP], and Cer PI-OH, were much more abundant in red and green algae (Supplementary Fig. 8 and Fig. 1B). However, the statistical analysis did not show clear clustering of the algae belonging to the Plantae kingdom (green and red algae) due to their more significant intra-group differences, as shown by the partitioning of carraghen and dulse algae in the PCA score plot (Supplementary Fig. 9A). The correlation heatmap confirmed the overall scarce correlation between the samples when the single lipids were employed as variables (Supplementary Fig. 9B). The inconsistency between the data shown in Fig. 1B and the statistical evaluation of the SL data matrix was investigated by building a data matrix in which the SP classes (the sum of the peak areas of the lipids belonging to each lipid class) were employed as variables (Supplementary Fig. 10), showing, as expected, two clusters of highly correlated SP classes (hydroxylated vs non-hydroxylated) as well as two clusters of highly correlated algae samples (brown vs red/green, PCC up to 0.8). Finally, a reduced data matrix comprising the sole 34 annotated free FAs furnished interesting results. Alongside PC2, in fact, two novel clusters can be observed, i.e., red algae and green/brown algae at negative and positive PC2 values, respectively (Supplementary Fig. 11A). Similar results were obtained by the hierarchical clustering (Supplementary Fig. 11B), which also showed an extremely scarce correlation in terms of the FA composition in the eight analyzed algae. The latter results could explain the inconsistency between the previous results obtained by using each single lipid or lipid class as variables. As such, some lipid classes seem to differentiate algae from the Plantae kingdom from brown algae, whereas free FA (and possibly conjugated FA also) followed completely different trends.

### Investigation of the fatty acyl composition of the annotated lipids

Based on the co-elution of diacyl lipids (GP and GL) bearing different pairs of fatty acyl chains and the inconsistency of the FA composition in the analyzed seaweeds, the lipidomics data from each of the eight analyzed samples were individually re-processed on Compound Discoverer to gather knowledge on the fatty acyl composition of the annotated lipids. For this purpose, a reduced lipid mass list that included the ID, molecular formulas, and molecular weight of the 543 annotated GL and GP sum compositions was employed, thus allowing faster data processing and manual interpretation of the MS/MS spectra. The latter approach differs significantly from the former which uses the “alignment” and “gap filling” tools to ensure that all peaks of the same molecular lipid from different runs are correctly aligned. This process exclusively regards MS1 data and enables the measurement of peak areas of molecular lipids whose MS1 spectrum is not associated with an MS2 spectrum. Moreover, in the case of missing peaks, the gap filling automatically inserts the noise level, thus avoiding missing or zero area values and allowing easier use of the resulting data matrix for statistical evaluations [48]. For these reasons, when subsets of data are analyzed individually, a significant reduction of the molecular lipids that can be annotated was expected, since several low-abundance peaks were not expected to be associated with an MS2 spectrum. As shown in Supplementary Fig. 12, the annotated GP ranged from 67 (dulse) to 208 (nori), corresponding to 22–63% of the number of GP annotated in the whole dataset, whereas the annotated GL ranged from 52 (dulse) to 138 (spaghetti algae), corresponding to 25–65% of the 212 annotated GLs from the whole dataset. Even more noteworthy, only 23 GPs and 22 GLs were annotated in each of the 8 analyzed seaweeds, corresponding to only 7% and 10% of the number of annotated molecular lipids, respectively. It is important to highlight that these results imply that there is a low number of lipids whose peak area is sufficient to trigger the MS/MS experiments that are needed for the lipid annotation, which is the main reason behind the need for simultaneous data processing of all samples, in which alignment and gap filling compensate for the limitation of the DDA data acquisition. Despite this, the data processing of the individual samples enabled the elucidation of the inter-species significant diversity in terms of fatty acyl composition. This is a crucial step when the lipidomes of heterogeneous samples are simultaneously analyzed, whereas it is not necessary for homogeneous samples, such as cell lipidomics [49] or our previous study on hempseeds from nine different strains [33]. It is also worth mentioning that a dual data acquisition would have been even more necessary in case HILIC separation was employed, giving that the latter separates lipids based on their polar heads [50]. In such conditions, not only SL isomers would likely co-elute, but the limitations of DDA would also be much more significant. On the other hand, the data processing of all samples cannot be bypassed, since it benefits from the complete feature alignment and gap filling that mitigate the limitations of DDA, thus furnishing a more thorough picture of the actual lipidome composition of the samples in study. Data-independent acquisition (DIA), in which all ions in a selected m/z window are simultaneously fragmented, would theoretically be helpful in bypassing the limitations of DDA for low-abundance lipids but suffers from poor performance with relatively slow Orbitrap instrumentation and would need a dedicated approach for MS/MS spectral deconvolution [51, 52].

**Fig. 2:**
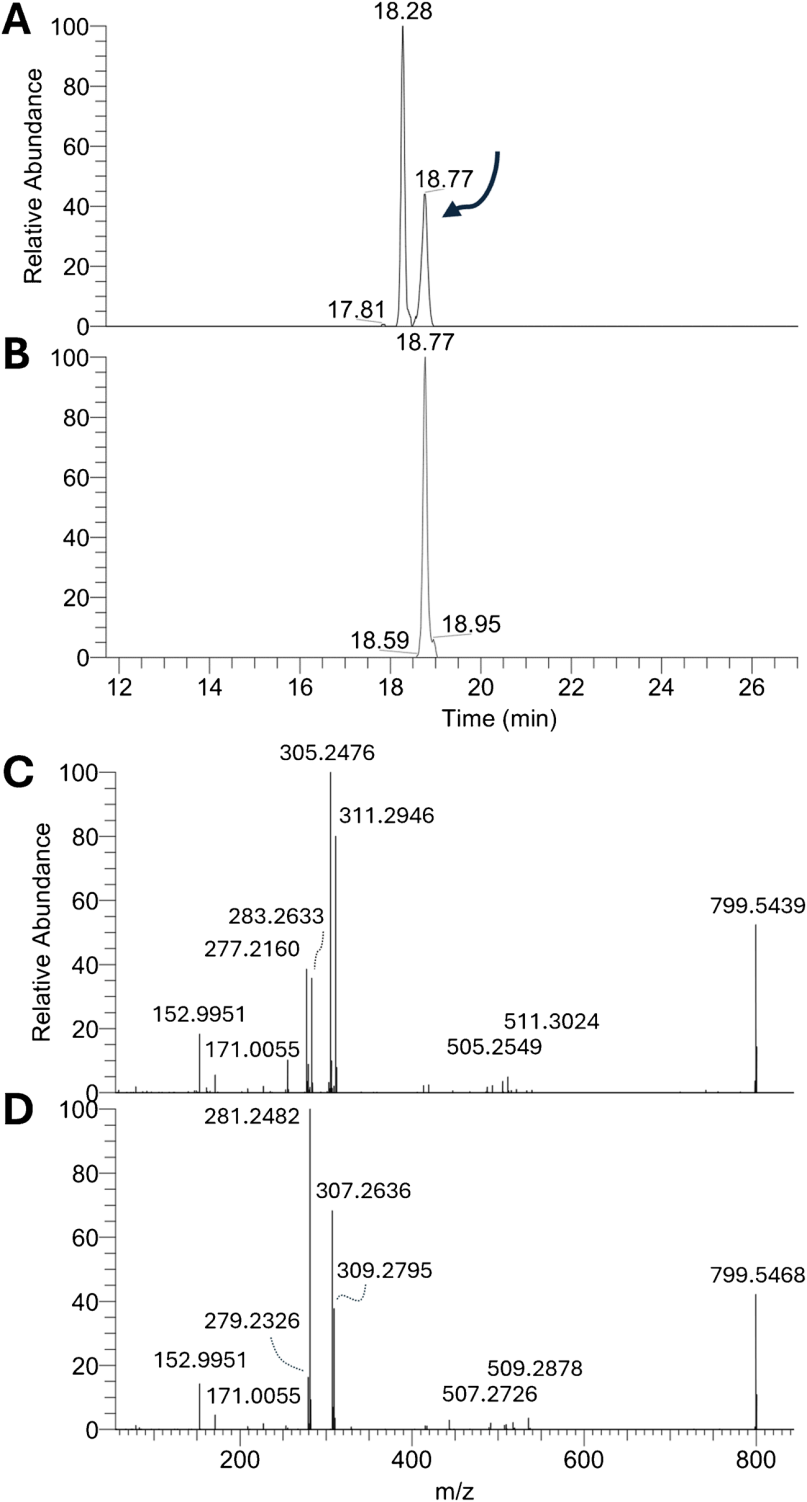
Extracted ion chromatograms (XICs) of the m/z corresponding to PG 38:3 from wakame (**A**) and sea lettuce (**B**) lipid extract analyses. MS/MS spectra associated with PG 38:3 from wakame (**C**) and sea lettuce (**D**) that show a fatty acyl composition corresponding to PG 18:0_20:3 + PG 18:3_20:0 and PG 18:1_20:2 + PG 18:2_20:1, respectively

The annotated fatty acyl compositions are reported for each analyzed seaweed in Supplementary Tables 2 and 3 for GP and GL, respectively. Except for a few exceptions, such as PI 34:1, which corresponded to the single isomer PI 16:0_18:1 for all analyzed seaweeds, most molecular lipids generated peaks corresponding to more than one pair of FA. Figure 2 shows the extracted ion chromatogram and associated MS/MS spectrum of PG 38:3 from brown algae wakame and green algae sea lettuce. Despite the exact same retention time (18.77), the fatty acyl composition was completely different in the two samples, and four distinct isomers were effectively hidden under the same aligned peak. Other examples of molecular lipids with the same retention properties under reversed-phase (RP) and different FA compositions are shown in Supplementary Fig. 13. A total of 506 different GPs with knowledge of their fatty acyl compositions were annotated, with an increase of around 53% from the 331 GP molecular lipids, whereas the increase was more restrained for GL (around 30%). These results confirmed the extreme variability of GP among the analyzed seaweeds that was observed earlier (Supplementary Figs. 3 and 4). Once the fatty acyl chain composition of the annotated diacyl lipids was investigated, the number of annotated lipids rose to 918.

### Annotation of lipid regioisomers

Once lipid sum composition and molecular lipids with information on the fatty acyl composition were annotated using the underivatized datasets and the two described data processing workflows, the derivatized lipid datasets were investigated to evaluate the regioisomeric distribution of carbon–carbon double bonds in free and conjugated FAs. For this purpose, lipid extracts were derivatized using the aPB reaction, a photochemical [2 + 2] cycloaddition between the double bonds of FA and the imine group of the derivatizing agent (6-AU). The aPB reaction was previously proposed by our research group [27] as an alternative to PB reactions for negative ion mode data acquisition and HCD fragmentation. Each lipid double bond generates two major reaction products with azetidine rings that undergo a characteristic that allows pinpointing the original position of the carbon–carbon double bond (Supplementary Fig. 14A). For example, aPB-derivatized FA 18:1 isomers with the double bond in omega-9 (ω-9) (oleic acid, OA) and omega-7 (ω-7) (vaccenic acid, VA) generate completely different/MS spectra with two diagnostic product ions (Supplementary Fig. 14B, C). For the other FAs, the double bond position was determined with the same rationale, keeping in mind that two diagnostic ions are generated for each double bond position and that multiply derivatized lipids had negligible peak area possibly due to the steric hindrance of the derivatizing reagent. To perform relative quantitation of the lipid regioisomers, the linear regressions obtained in our previous study [27] for ω-9/ω-7 and ω-6/ω-3 were employed. As such, we demonstrated that, despite the ion intensities cannot be directly compared because they are derived from different structures and are triggered on slightly different positions of the peak, their ion ratios can be associated with molar ratios in line with what was previously demonstrated for PB reactions [25]. Therefore, ω-9/ω-7 and ω-6/ω-3 ratios were rigorously calculated, whereas other regioisomers could only be estimated based on the ion intensities. The complementary results obtained by the underivatized and derivatized datasets allowed the elucidation of the FA composition of the analyzed seaweed samples (Table 1). As expected, the relative abundances of the single FA were significantly diversified among the eight analyzed seaweeds. FA 16:0, for example, ranged from 5.6% (wakame) to 40% (carraghen), while FA 20:4 ranged from 0.7% (carraghen) to 29% (nori). More even results were obtained when FAs were grouped based on their saturation into SFA, MUFA, and PUFA. As such, with the notable exception of carraghen, PUFAs were the most abundant FA in all other samples, comprising up to 83% of the total FA peak area, whereas SFA and MUFA were evenly distributed (9.8–22% and 7–35%, respectively) in good agreement with previous studies [53–57]. The annotation of the MS/MS spectra of the aPB-derivatized FA revealed several regioisomers of FA hidden under the same peak. Given the conversion of the aPB reactions, the MS/MS spectra could only be obtained for underivatized peaks that were at least ten times more intense than the noise level.

**Table 1:** Fatty acid composition of the eight analyzed seaweed species after B&D extraction and HRMS analysis

	Kombu	Saccharina	Spaghetti	Wakame	Sea lettuce	Carraghen	Dulse	Nori
	*Laminaria digitata*	*Saccharina latissima*	*Himanthalia elongata*	*Undaria pinnatifida*	*Ulva lactuca*	*Chondrus crispus*	*Palmaria palmata*	*Porphyra umbilicalis*
	Brown	Brown	Brown	Brown	Green	Red	Red	Red
	%	%	%	%	%	%	%	%
FA 14:0	1.06 ± 0.05	2.8 ± 0.1	1.69 ± 0.01	0.41 ± 0.01	0.3 ± 0.1	2.2 ± 0.1	1.8 ± 0.2	0.08 ± 0.01
FA 16:0	13.2 ± 0.9	10.7 ± 0.3	11.5 ± 0.7	5.6 ± 0.3	12 ± 3	40 ± 5	13.1 ± 0.8	6.9 ± 0.6
FA 16:1	7.1 ± 0.4	5 ± 1	2.3 ± 0.1	0.74 ± 0.03	3.3 ± 0.6	4 ± 1	4.0 ± 0.3	0.43 ± 0.04
FA 18:0	2.6 ± 0.2	1.87 ± 0.05	1.22 ± 0.03	1.34 ± 0.05	1.2 ± 0.3	9.4 ± 0.3	2.9 ± 0.1	1.6 ± 0.1
FA 18:1	19.8 ± 0.9	20.2 ± 0.6	17.0 ± 0.8	5.9 ± 0.1	18 ± 2	19 ± 2	15.5 ± 0.5	7.4 ± 0.6
FA 18:2	11.0 ± 0.6	7.9 ± 0.3	7.1 ± 0.4	7.1 ± 0.2	8 ± 2	1.3 ± 0.1	5.7 ± 0.2	2.2 ± 0.2
FA 18:3	4.4 ± 0.5	4.4 ± 0.3	12.7 ± 0.6	12 ± 1	10 ± 2	0.5 ± 0.1	4.6 ± 0.4	0.02 ± 0.01
FA 18:4	5.0 ± 0.1	8.1 ± 0.2	9.1 ± 0.2	19 ± 1	22 ± 3	0.2 ± 0.1	5.5 ± 0.4	0.14 ± 0.01
FA 20:0	0.8 ± 0.1	1.5 ± 0.1	0.72 ± 0.04	0.95 ± 0.04	0.5 ± 0.1	0.8 ± 0.1	0.76 ± 0.05	0.03 ± 0.01
FA 20:1	0.39 ± 0.02	0.5 ± 0.1	0.11 ± 0.01	0.03 ± 0.01	0.6 ± 0.1	6.4 ± 0.2	1.66 ± 0.04	6.2 ± 0.6
FA 20:2	1.0 ± 0.1	0.54 ± 0.05	0.59 ± 0.01	0.09 ± 0.01	0.2 ± 0.1	1.7 ± 0.2	0.6 ± 0.1	2.9 ± 0.3
FA 20:3	0.58 ± 0.03	1.7 ± 0.1	2.22 ± 0.05	1 ± 0.1	0.8 ± 0.1	0.17 ± 0.01	0.60 ± 0.04	7.4 ± 0.3
FA 20:4	18.3 ± 0.6	19.9 ± 0.8	20.2 ± 0.6	22 ± 1	1.9 ± 0.4	0.7 ± 0.1	7.9 ± 0.4	29 ± 5
FA 20:5	8.5 ± 0.2	11.5 ± 0.4	8.2 ± 0.2	21.9 ± 0.6	2.7 ± 0.3	1.0 ± 0.3	23 ± 2	33 ± 1
Others	6.2 ± 0.4	3.7 ± 0.2	5.3 ± 0.3	2.1 ± 0.2	18 ± 3	12 ± 1	13 ± 1	2.9 ± 0.3
SFA	19 ± 1	18.2 ± 0.7	19 ± 1	9.8 ± 0.5	21 ± 4	59 ± 5	22 ± 1	8.8 ± 0.7
MUFA	32 ± 2	26 ± 2	20.3 ± 0.9	7.0 ± 0.2	24 ± 4	35 ± 3	29 ± 1	16 ± 1
PUFA	49 ± 2	55 ± 2	60 ± 2	83 ± 4	56 ± 9	6 ± 1	49 ± 4	76 ± 7
ω6	29 ± 1	27 ± 1	27.1 ± 0.9	29 ± 1	9 ± 2	3.7 ± 0.4	13.6 ± 0.7	40 ± 5
ω3	18.4 ± 0.8	24.9 ± 0.9	30.2 ± 0.9	53 ± 3	44 ± 6	1.5 ± 0.3	33 ± 3	34 ± 6
OA/VA	5.4 ± 0.3	94 ± 10	70 ± 7	n.a	0.16 ± 0.02	2.7 ± 0.1	2.2 ± 0.3	3.4 ± 0.3
ALA/GLA	n.a	7 ± 1	n.a	n.a	33 ± 9	n.a	15 ± 3	n.a
FA 20:4 ω6/ω3	14 ± 5	11.0 ± 0.7	70 ± 6	77 ± 6	0.10 ± 0.04	n.a	24 ± 3	27 ± 8
PUFA/SFA	2.6 ± 0.2	3.0 ± 0.2	3.2 ± 0.3	9.2 ± 0.9	2.7 ± 0.9	0.10 ± 0.03	2.2 ± 0.3	9 ± 1
ω6/ω3	1.6 ± 0.1	1.1 ± 0.1	0.9 ± 0.1	0.55 ± 0.05	0.20 ± 0.07	2.5 ± 0.8	0.41 ± 0.06	1.2 ± 0.4

OA/VA, ALA/GLA, and FA 20:4 ω6/ω3 ratios were calculated by comparison of the diagnostic ions obtained following aPB derivatization and MS/MS fragmentation. The % were estimated based on the peak area of the non-derivatized FA peaks

**Fig. 3:**
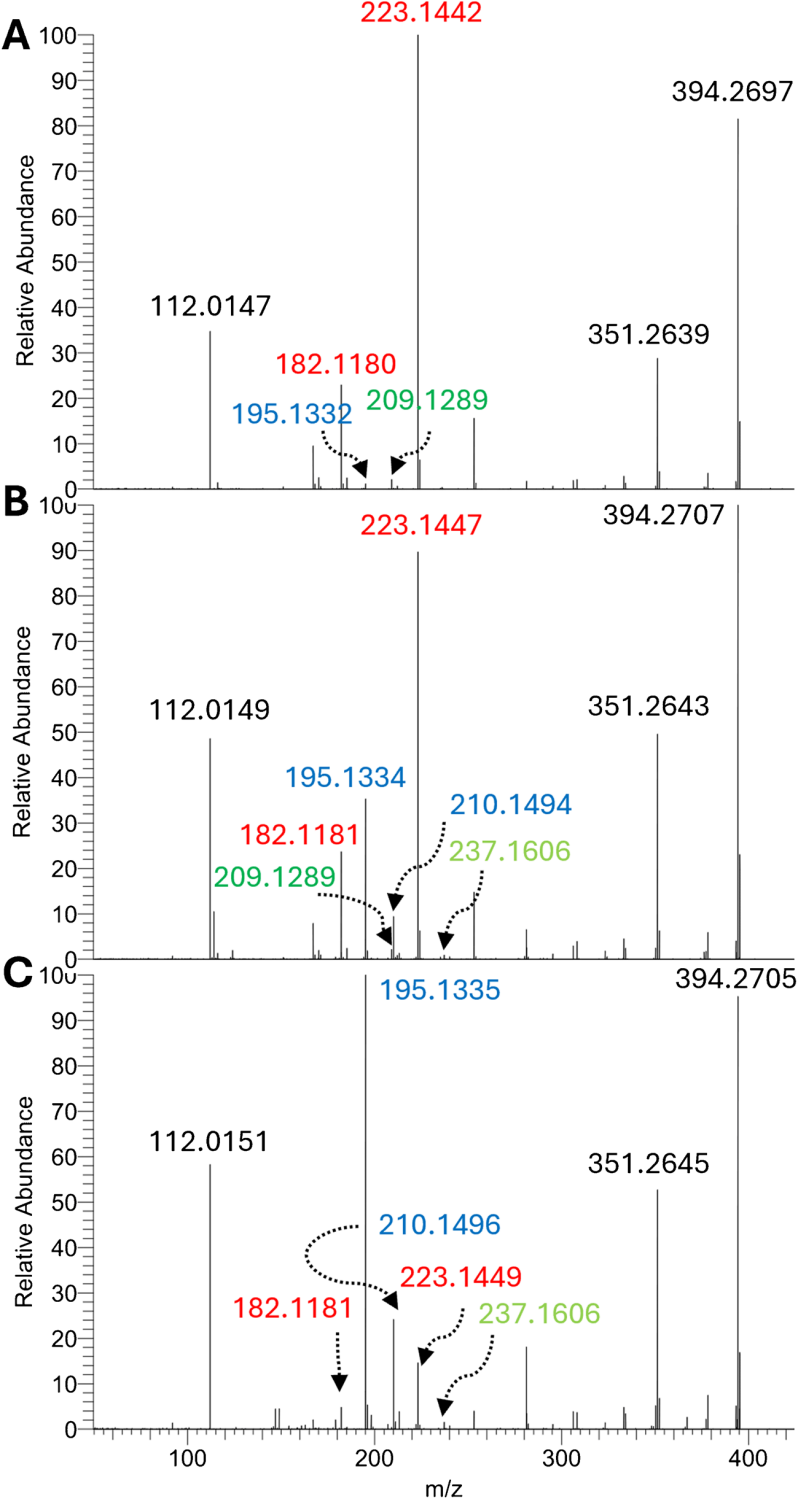
MS/MS spectra associated with aPB-derivatized FA 18:1 from brown algae saccharina (**A**), red algae dulse (**B**), and green algae sea lettuce (**C**). Diagnostic product ions for ω-7, ω-8, ω-9, and ω-10 are marked in blue, dark green, red, and light green, respectively

**Fig. 4:**
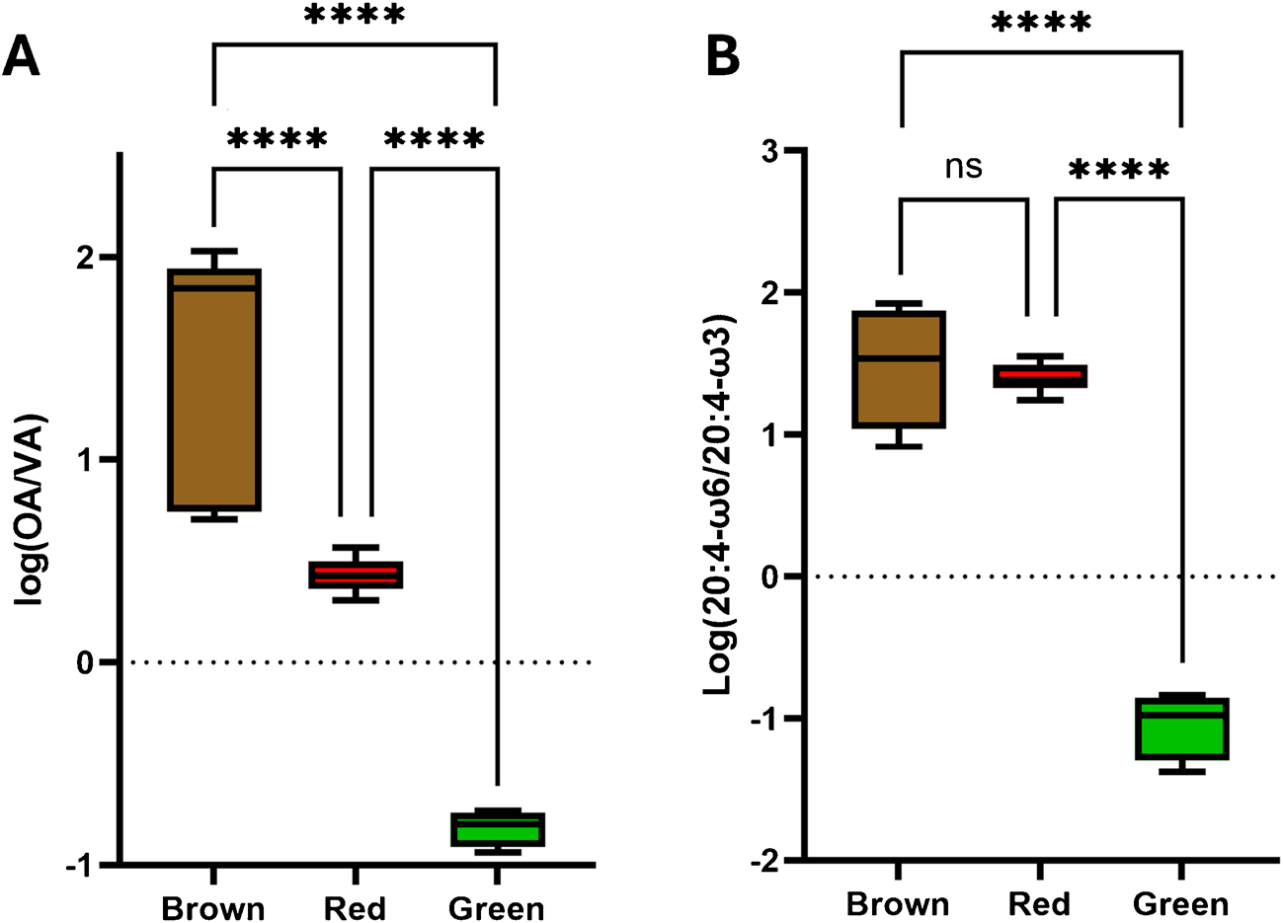
Box and whisker plots displaying the OA/VA ratio (**A**) and ω-6/ω-3 ratio of FA 20:4 regioisomers (**B**) in the analyzed brown, red, and green seaweed samples after aPB derivatization and LC–MS analysis; *t*-test analysis was performed: ^ns^*p* > 0.1 and ****p < 0.0001

The diagnostic product ions as well as the relative abundances of FA regioisomers are reported in Supplementary Table 1. Four isomers of FA 16:1 were annotated, i.e., ω-3, ω-5, ω-7, and omega-8 (ω-8), with ω-7 being by far the most abundant in all samples whose peak area was sufficient for the obtention of the MS/MS spectrum (with an estimated abundance in the range of 87.9–100% based on the ion intensities). Similarly, four isomers of FA 18:1 were annotated, i.e., ω-7, ω-8, ω-9, and omega-10 (ω-10), and all algae (except for wakame) had at least two distinct regioisomers (Fig. 3). Notably, sea lettuce stood out from all other seaweeds for its prevalence of the ω-7 isomer (VA) over the most common ω-9 (OA). As expected, FA 18:3 was a mixture of ω-3 (ALA) and ω-6 (γ-linolenic acid, GLA) isomers, with the former being more prevalent, whereas FA 18:2 and FA 18:4 were exclusively ω-6 and ω-3 in all analyzed samples, respectively. The regioisomerism of FA 20:1 could only be evaluated for carraghen, resulting in a prevalence of the ω-7 over the ω-9 isomer, and nori, which was exclusively constituted by the ω-9 isomer (Supplementary Fig. 15). FA 20:2 was also characterized for the sole carraghen and nori (100% ω-6) and FA 20:3 had sufficient peak area only in nori (100% ω-6). In analogy with FA 18:1, sea lettuce stood out from all other seaweeds because of having a prevalence of the ω-3 regioisomer of FA 20:4 over the more common ω-6 (arachidonic acid) (Supplementary Fig. 16). Conversely, FA 20:5 was a single regioisomer (ω-3) for all analyzed seaweeds. Finally, FAs 22:5 and 24:1 were sufficiently abundant solely in sea lettuce (100% ω-3) and dulse (100% ω-9), respectively. The global ω-6/ω-3 ratio, which is the ratio of the diagnostic ion characteristics of such isomers in all FA MS/MS spectra, was the lowest in green algae sea lettuce (0.2), whereas it was around 1 for most other analyzed seaweeds, in good agreement with previously obtained results with other analytical techniques [53, 56]. However, compared to previous results [53], the measured ω-6/ω-3 for sea lettuce appeared significantly lower. These inconsistencies could be due to an incorrect evaluation of the regioisomerism of FA 20:4, which our results demonstrated as being mainly the uncommon ω-3 isomer. As such, previous GC–MS results on *Ulva lacinulata*, another seaweed of the family Chlorophyceae, demonstrated that FA 20:4 ω-3 was the main isomer and measured ω-6/ω-3 in the range 0.15–0.30 [58]. The most interesting results were relative to the ratios between the two main isomers of FA 18:1 (ω-9 vs ω-7) and FA 20:4 (ω-6 vs ω-3) in relation to the class of analyzed seaweeds. The ratios among these two pairs of regioisomers are shown in the box and whisker plots shown in Fig. 4 in a logarithmic scale to visualize and compare values in a range of 3 orders of magnitude.

The measured values per algae class were then analyzed by *t*-test analysis (brown vs red, brown vs green, and red vs green), as shown in Fig. 4. As such, the OA/VA ratio appeared to statistically discriminate all three classes of seaweeds (Fig. 4A), with brown algae showing a high prevalence of the ω-9 isomer (OA/VA > 5), red algae with an OA/VA ratio around 2–3, and green algae with a prevalence of the ω-7 isomer (OA/VA < 1). On the other hand, the ω-6/ω-3 ratio of FA 20:4 isomers did not discriminate brown vs red seaweeds (ω-6/ω-3 > 10), but, as mentioned earlier, allowed sea lettuce to stand out (ω-6/ω-3 < 1; Fig. 4B). Further studies with a higher number of different seaweed samples (especially green macroalgae) are needed to confirm these results, despite previous studies by GC appearing to confirm the peculiarity in the ω-6/ω-3 ratio of FA 20:4 isomers in green seaweeds [58].

Finally, the regiochemistry of carbon–carbon double bonds of conjugated FA was evaluated. In this study, for the first time, the aPB reaction was employed for pinpointing carbon–carbon double bonds in GL, thus confirming the versatility of the reaction, which reacts with free and conjugated FAs regardless of the polar head of the lipids. As discussed in the section “Annotation of lipid regioisomers,” the peak areas of molecular lipids of GP and GL as well as their fatty acyl composition were extremely inconsistent in the eight analyzed seaweeds. As such, considering the need for peak areas at least ten times higher than the noise level for the aPB derivatives to be fragmented, few GPs and GLs could effectively be compared in terms of their carbon–carbon double bond regiochemistry. Not unexpectedly, the higher the degree of structural detail that is searched for, the lower the number of lipids that can be annotated and compared. Supplementary Table 5 lists the regiochemistry assignment of 18 lipid sum compositions whose aPB derivatives could be annotated in at least 3 different seaweeds. Not unexpectedly, the regioisomerism of conjugated FA mirrored that of free FA, e.g., the 18:1 chain of DGDG 34:1 had a prevalence for the ω-9 regioisomer in brown and red seaweeds and a prevalence for the ω-7 regioisomer in sea lettuce samples. The only major exception was represented by an abnormally high abundance of the ω-8 isomer of FA 18:1 in PG 34:1 and PI 34:1 (but not SQDG 34:1 and DGDG 34:1).

## Conclusions

Edible seaweeds are emerging as novel foods for their rich content of macronutrients, micronutrients, and bioactive compounds towards the alleviation of risk factors associated with the metabolic syndrome. Despite in small overall quantity, the lipid content of seaweeds has raised significant interest due to the high abundance of bioactive and essential PUFAs. In lipidomics analysis, the choice of the analytical platform significantly affects the performance of the method in terms of the number of annotated lipids and the degree of structural detail in their annotation. Data processing of highly diversified samples, moreover, was proven to hide possible obstacles in the correct annotation of the molecular lipid species. As such, despite separating lipids based on their fatty acyl chains, RP was proven often incapable of separating lipid isomers that differ for their fatty acyl composition, not to mention regioisomers with different carbon–carbon double bond locations. Therefore, when samples with highly diversified lipidomes are analyzed simultaneously, as in the case of seaweeds, the data processing workflows that align peaks fill the gaps among the runs, and associate MS/MS to features that could lead to incorrectly annotating the identity of co-eluting lipid isomers among the different samples. Based on these findings, a triple-data processing strategy was carried out to achieve high structural detail on seaweed lipidome to annotate lipid sum compositions, molecular lipids, and lipid regioisomers, respectively. Our results demonstrated that the lower the structural detail of the annotated lipidome, the broader the number of lipids that can be annotated and compared between samples. Conversely, a high level of structural detail often leads to the impossibility of comparing samples with significantly different lipidomes. Seaweeds were a perfect example of matrices that are often analyzed together but have significant differences in their lipidomes, thus requiring careful data processing to compare the samples and achieve high structural detail. The results gathered from such detailed lipid data processing enabled to fill the gap in the knowledge of the lipidome of edible seaweeds and to explore unknown differences in the regioisomer composition of seaweeds from different families, such as the OA/VA and FA 20:4 ω-6/ω-3 ratios. Further studies are needed to confirm the results to expand seaweed species datasets and to determine if the biological activities of the lipid extract could be correlated with the differential composition in FA regioisomers.

## Supplementary information

Below is the link to the electronic supplementary material.

Supplementary file1 (DOCX 2607 KB)

Supplementary file2 (XLSX 100 KB)

Supplementary file3 (XLSX 348 KB)
