# Supplementary material for: Detailed lipid investigation of edible seaweeds by photochemical derivatization and untargeted lipidomics
Source: Anal Bioanal Chem. 2024 Oct 11;416(28):6269–82. doi: 10.1007/s00216-024-05573-6 (PMC11541411; doi:10.1007/s00216-024-05573-6)
Supplement: Supplementary file 1 — Supplementary file1 (DOCX 2607 KB) [file 216_2024_5573_MOESM1_ESM.docx]

**Supplementary Materials – Word File**

**Detailed lipid investigation of edible seaweeds by photochemical derivatization and untargeted lipidomics**

Carmela Maria Montone^1^, Chiara Cavaliere^1^, Andrea Cerrato^1,*^, Aldo Laganà^1^, Susy Piovesana^1^, Enrico Taglioni^1^, Anna Laura Capriotti^1^

^1^ Department of Chemistry, Sapienza University of Rome, Piazzale Aldo Moro 5, 00185 Rome, Italy

*Corresponding author

Department of Chemistry

Università di Roma “La Sapienza”

Piazzale Aldo Moro 5

00185 Rome, Italy

E-mail: andrea.cerrato@uniroma1.it

tel: +39 06 4991 3945


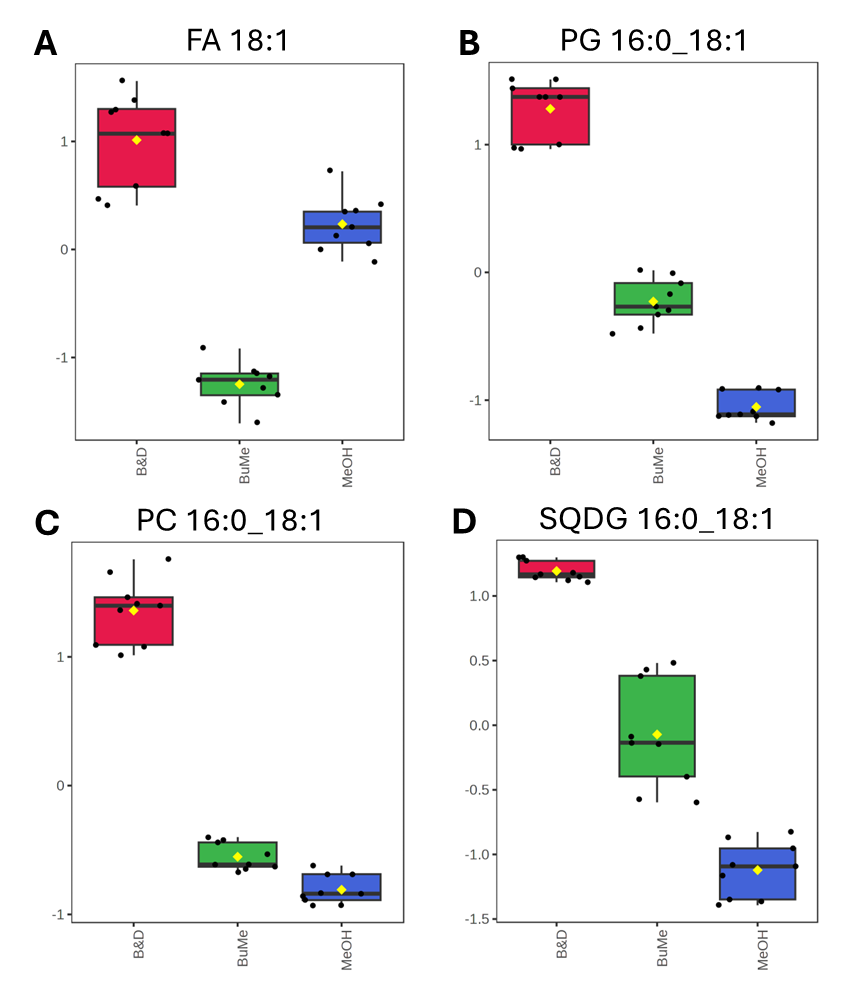


**Figure S1.** Box plots of four exemplary lipids extracted from the macroalgae pooled sample using the B&D extraction protocol in comparison to two (B&D, Matyash, BuMe, and MeOH).


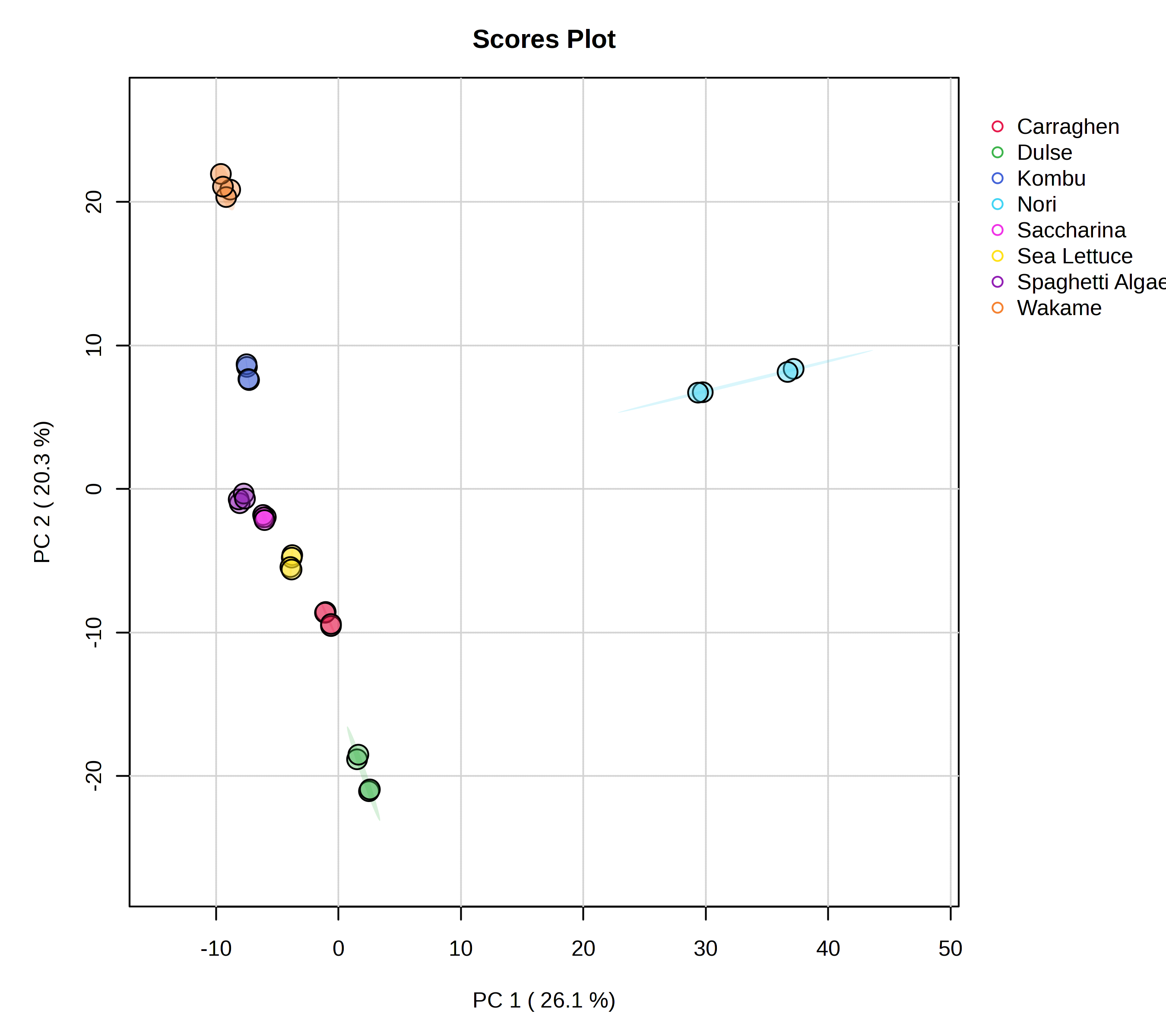


**Figure S2.** Principal component analysis (PCA) scores plot obtained using the data matrix of the 679 annotated lipid sum compositions in the 8 analyzed macroalgae.


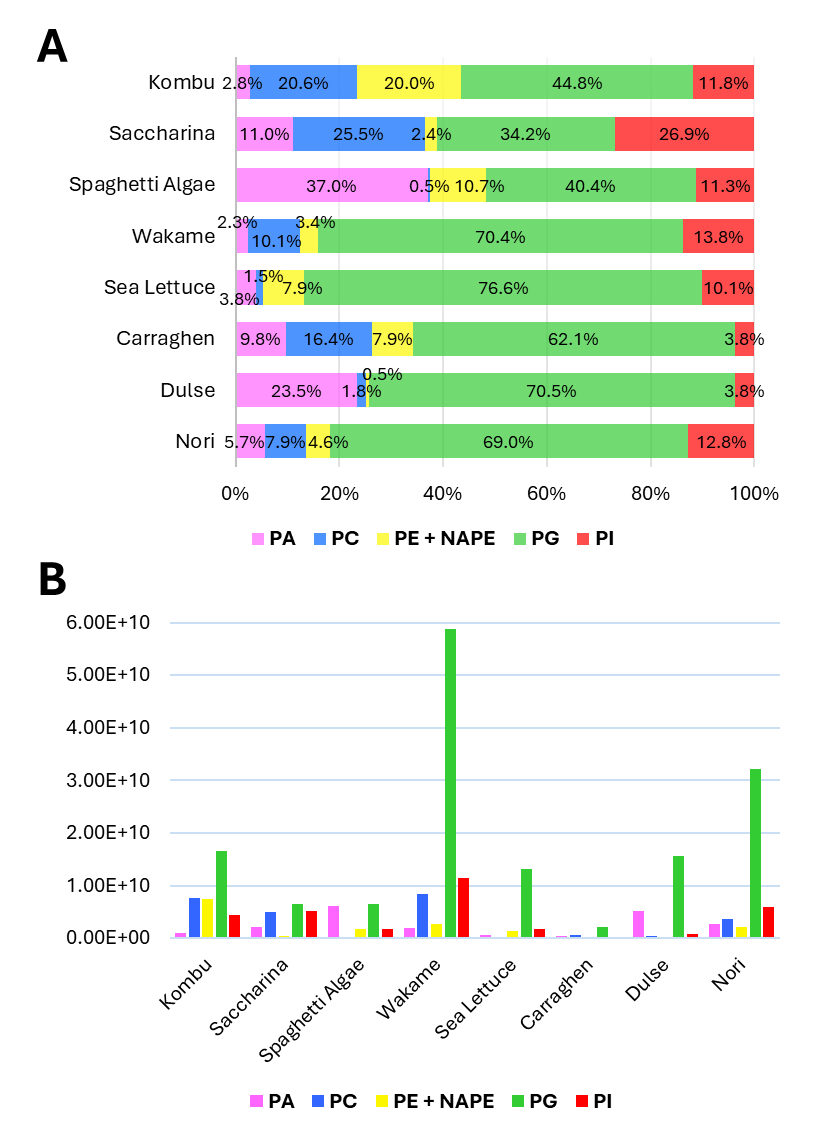


**Figure S3.** Stacked bar chart displaying the relative peak areas of the GP classes in the 8 analyzed macroalgae (A). Bar chart displaying the total peak area per GP class in the 8 analyzed macroalgae (B).


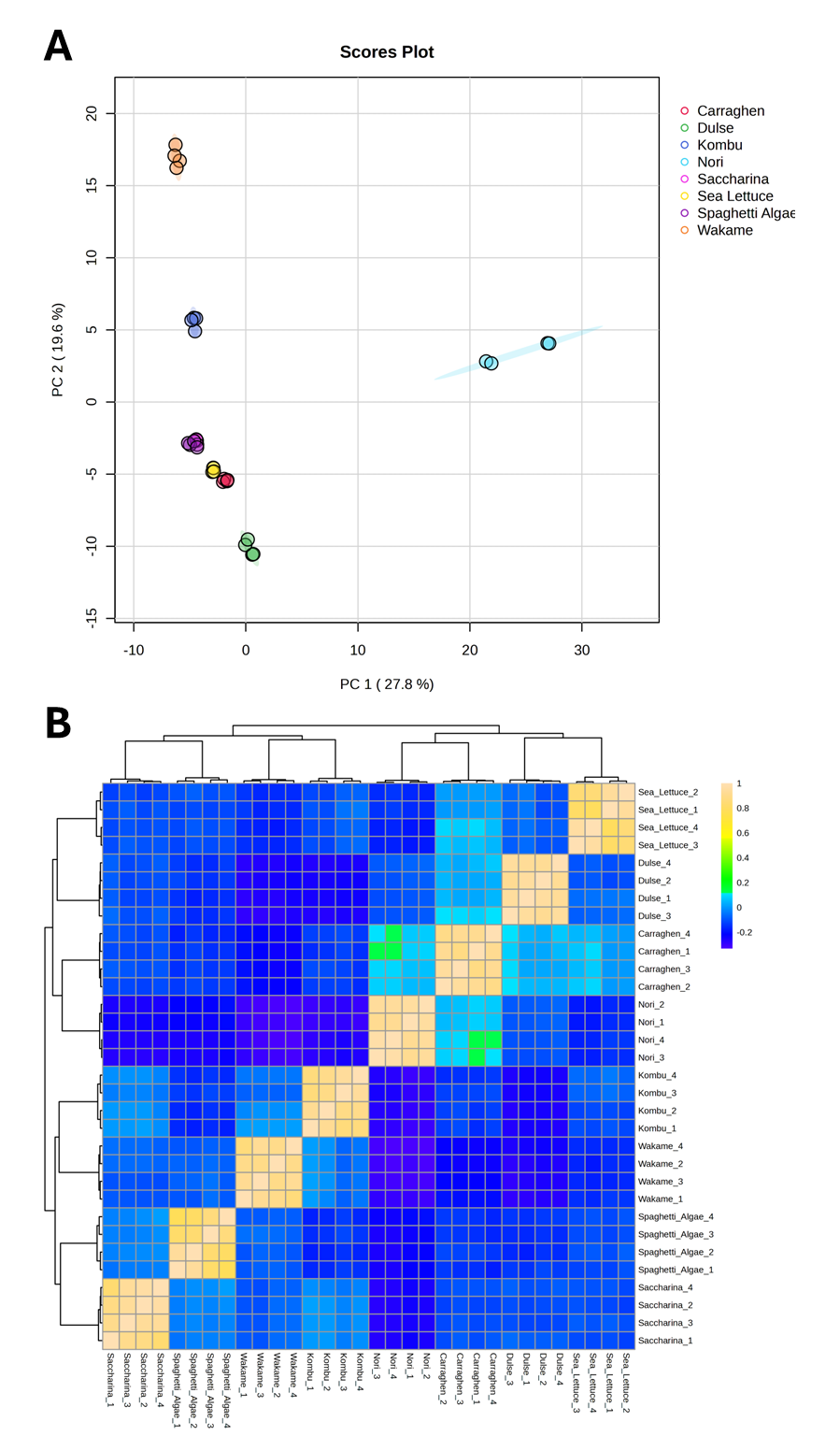


**Figure S4.** Principal component analysis (PCA) scores plot (A) and correlation heatmap hierarchical clustering (B) obtained using the 331 annotated GP data matrix in the 8 analyzed macroalgae.


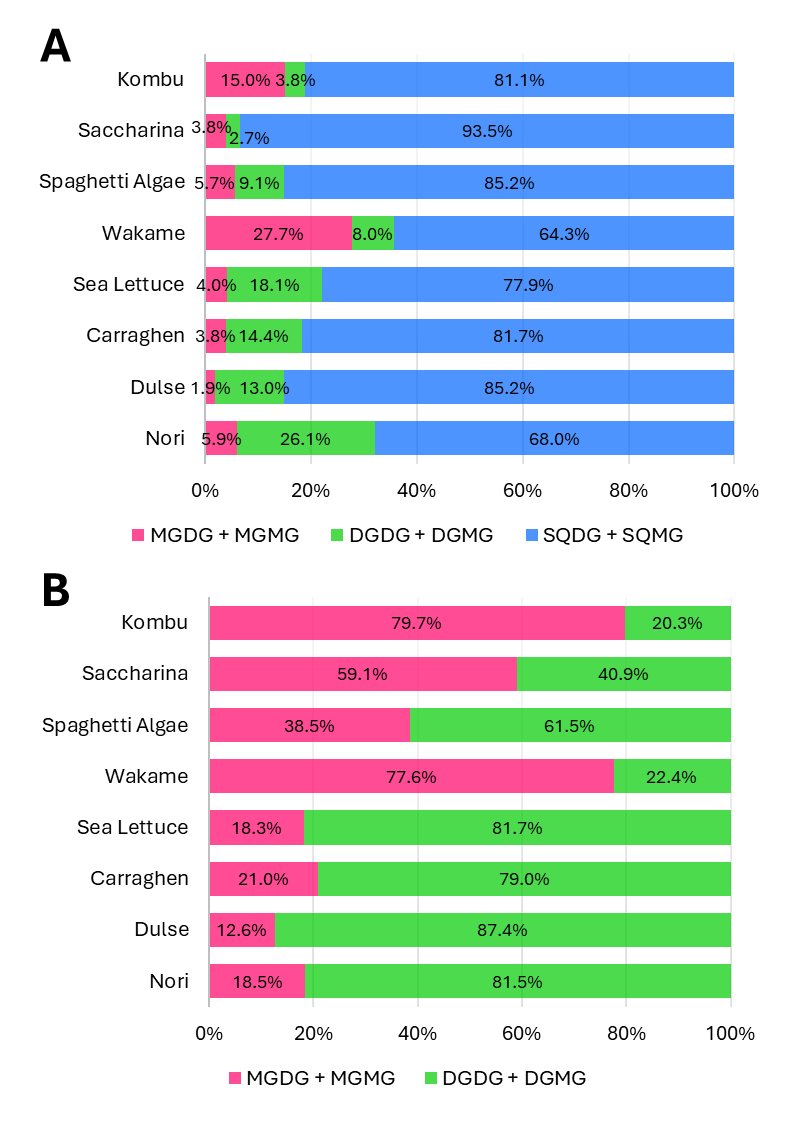


**Figure S5.** Stacked bar charts displaying the relative peak areas of the GL classes (A) and of MGDG + MGMG vs DGDG + DGMG (B) in the 8 analyzed macroalgae.


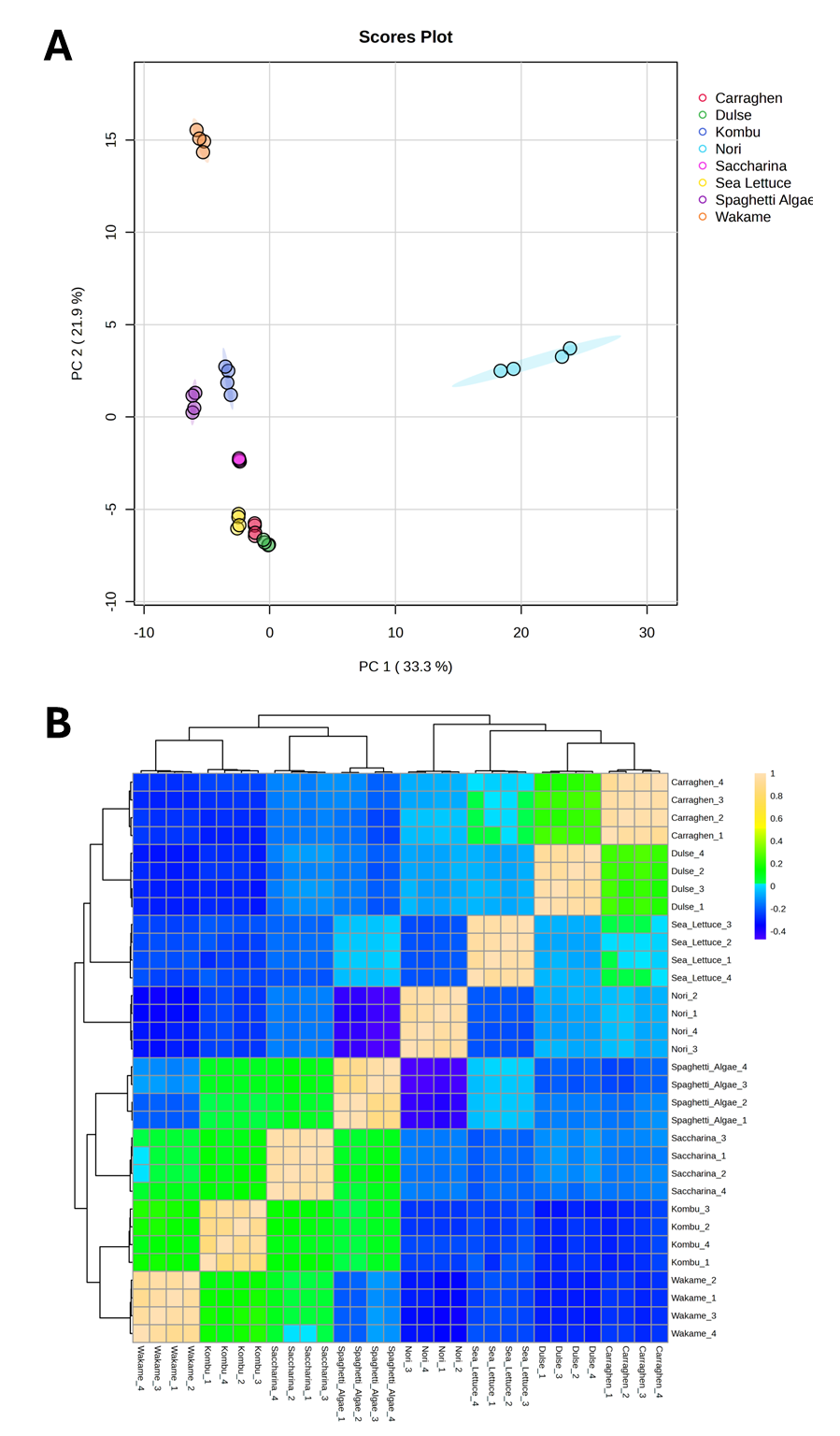


**Figure S6.** Principal component analysis (PCA) scores plot (A) and correlation heatmap hierarchical clustering obtained using the 212 annotated GL data matrix in the 8 analyzed macroalgae.


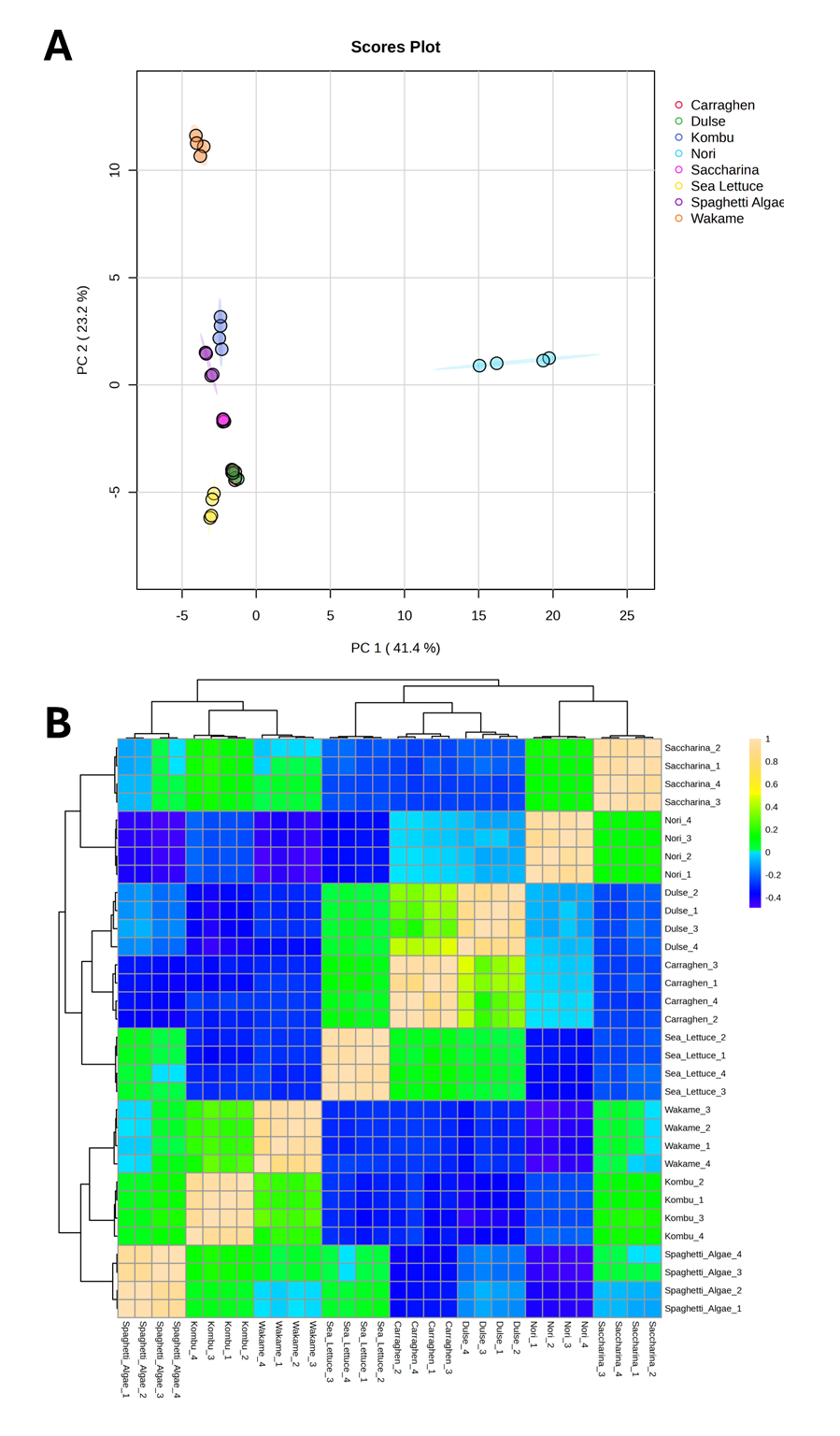


**Figure S7.** Principal component analysis (PCA) scores plot (A) and correlation heatmap hierarchical clustering obtained using the 113 annotated galactosyl lipids data matrix in the 8 analyzed macroalgae.


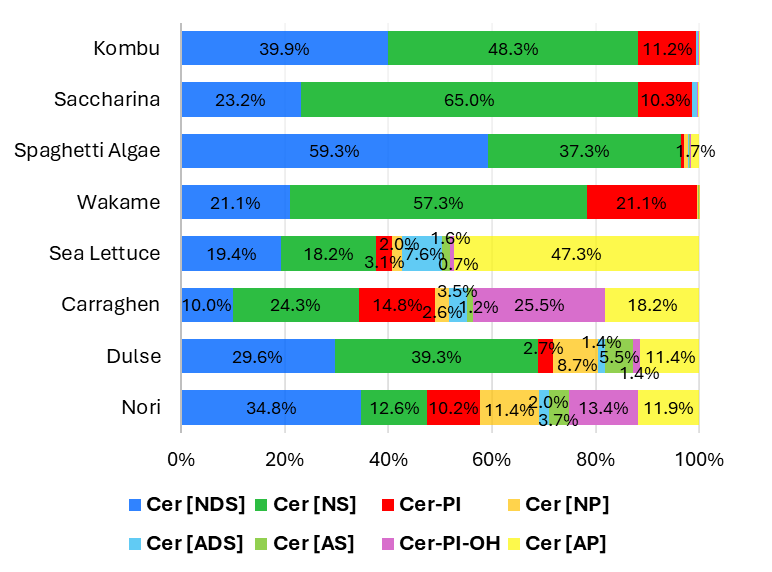


**Figure S8.** Stacked bar charts displaying the relative peak areas of the SP classes in the 8 analyzed macroalgae.


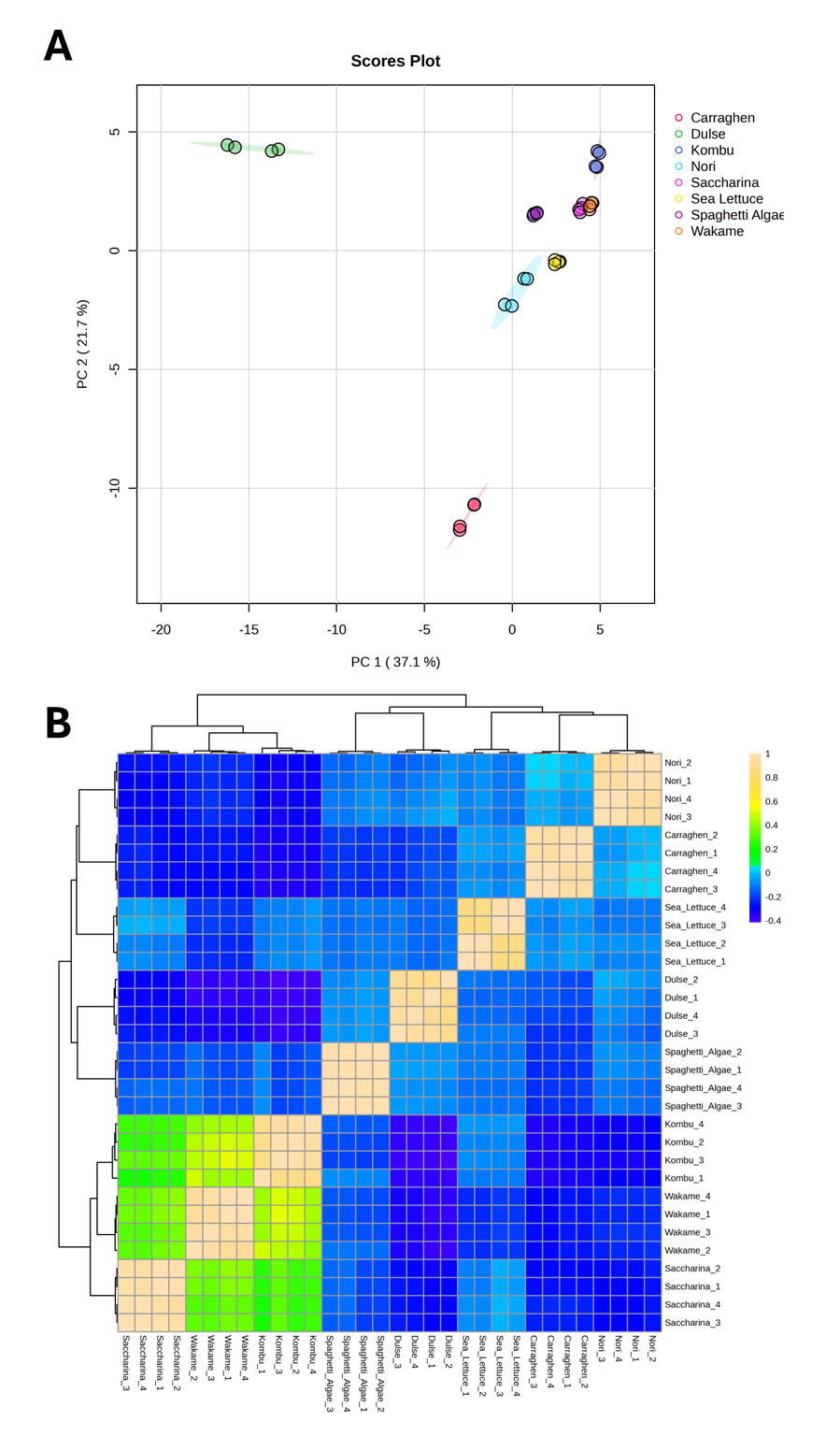


**Figure S9.** Principal component analysis (PCA) scores plot (A) and correlation heatmap hierarchical clustering obtained using the 102 annotated SP data matrix in the 8 analyzed macroalgae.


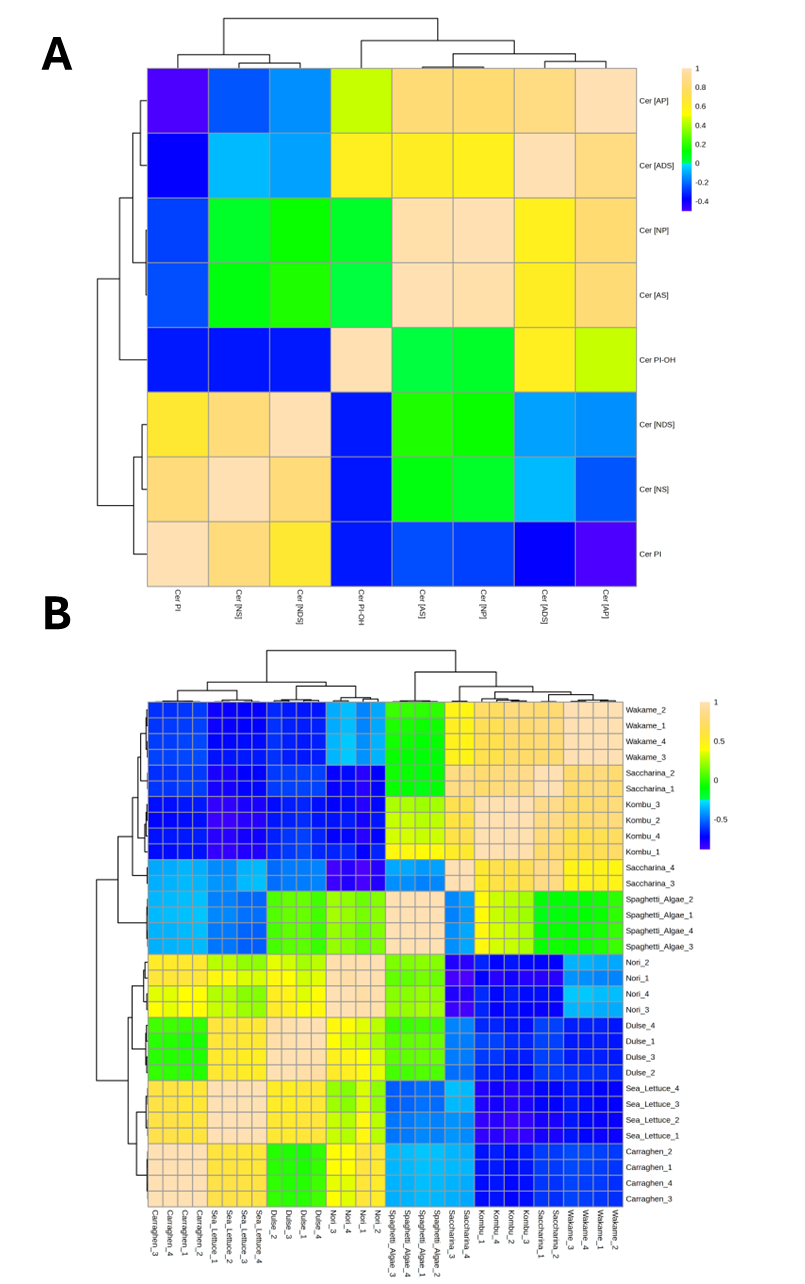


**Figure S10.** Correlation heatmap hierarchical clustering obtained using the 8 annotated SP classes data matrix in the 8 analyzed macroalgae, SL classes used as variables (A) and algae samples used as variables (B).


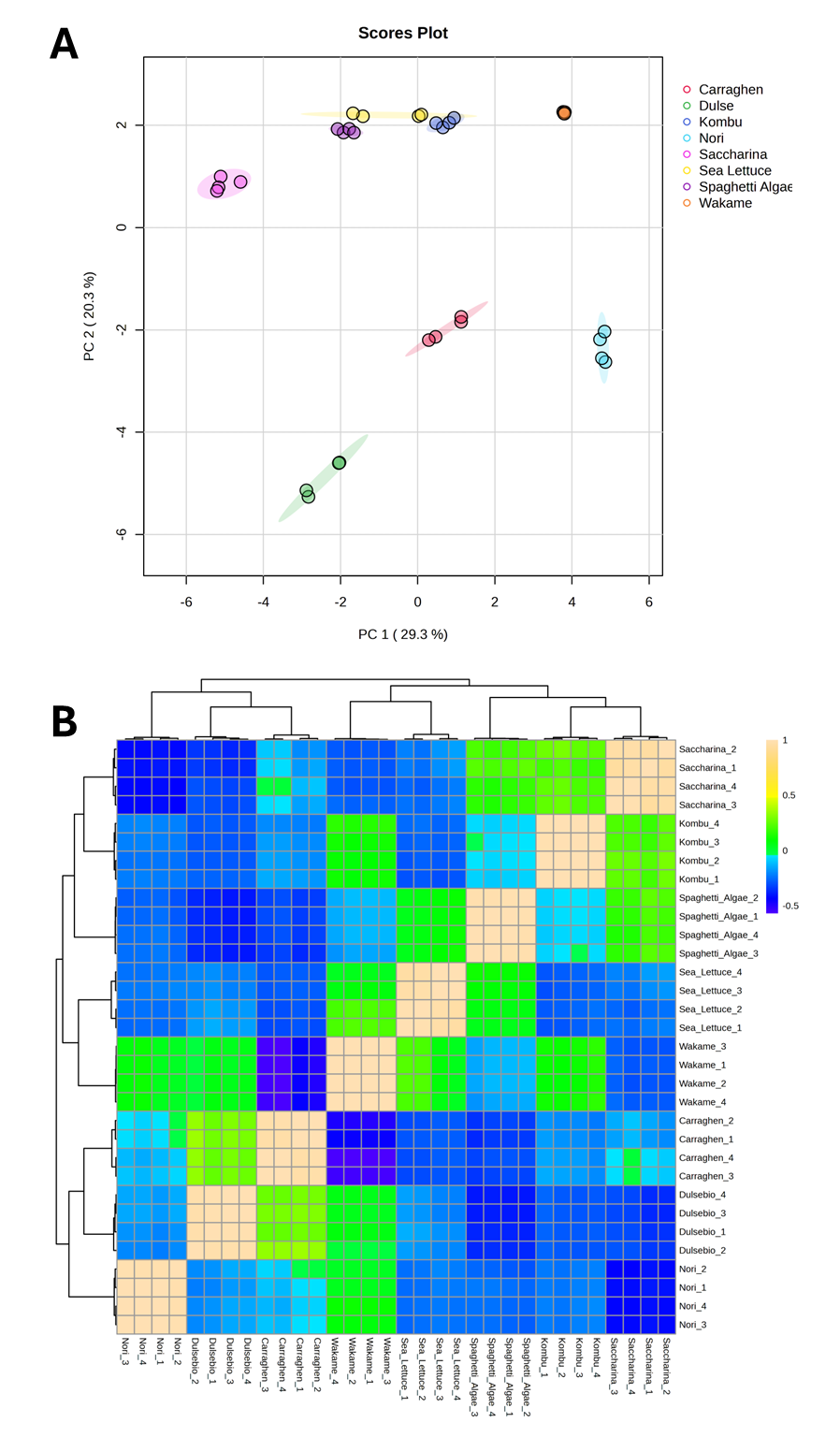


**Figure S11.** Principal component analysis (PCA) scores plot (A) and correlation heatmap hierarchical clustering (B) obtained using the 34 annotated FA data matrix in the 8 analyzed macroalgae.


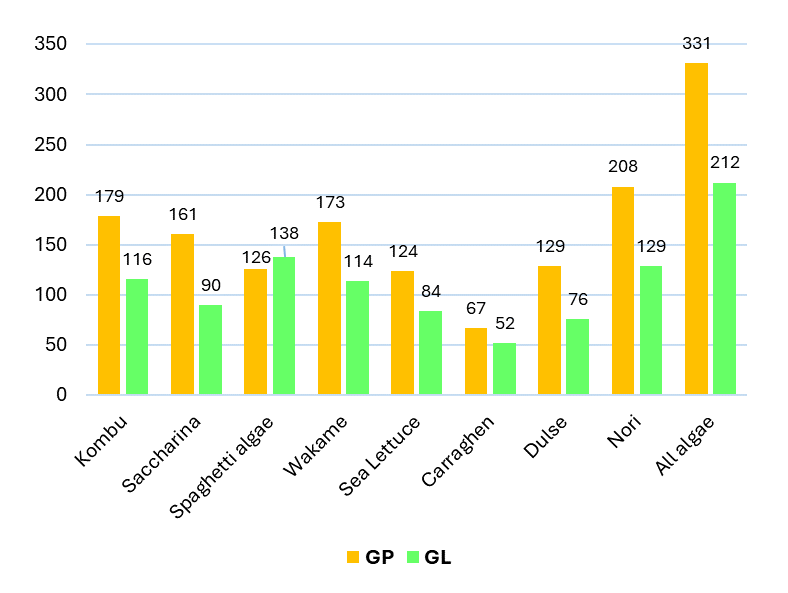


**Figure S12.** Bar chart of the GP and GL molecular lipids annotated from the data processing of each macroalgae analyzed individually compared to the results of all the lipid extracts data processed together.


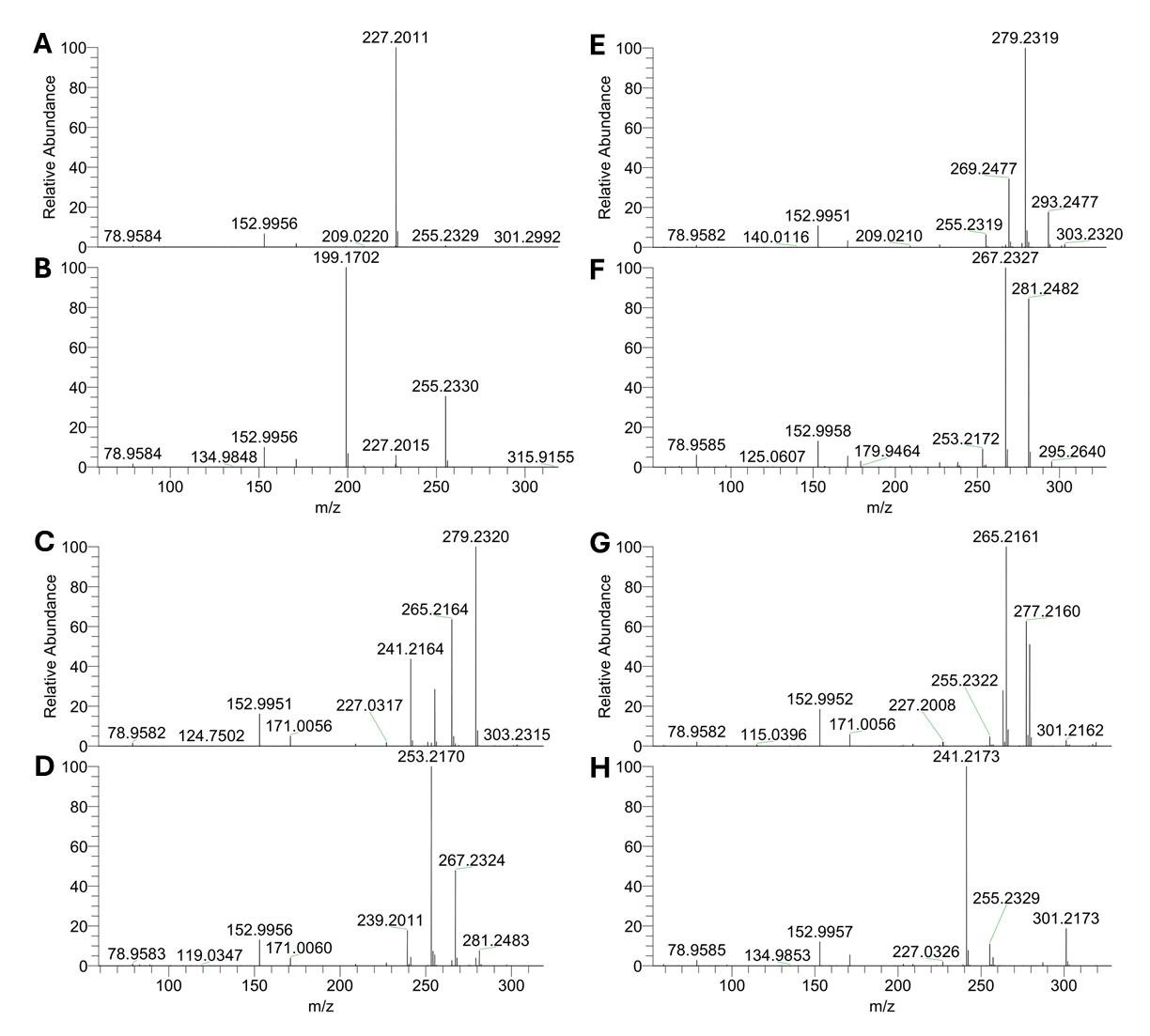


**Figure S13.** MS/MS spectra (30 NCE) associated with the m/z of PG 28:0 from Dulse (A) and Kombu (B), that show a fatty acyl composition corresponding to PG 14:0/14:0 and PG 12:0_16:0, respectively. MS/MS spectra (30 NCE) associated with the m/z of PG 33:2 from Wakame (C) and Sea Lettuce (D), that show a fatty acyl composition corresponding to PG 15:0_18:2 + PG 16:0_17:2 and PG 15:1_18:1 + PG 16:1_17:1, respectively. MS/MS spectra (30 NCE) associated with the m/z of PG 35:2 from Wakame (E) and Carraghen (F), that show a fatty acyl composition corresponding to PG 16:0_19:2 + PG 17:0_18:2 and PG 16:1_19:1 + PG 17:1_18:1, respectively. MS/MS spectra (30 NCE) associated with the m/z of PG 35:5 from Wakame (G) and Nori (H), that show a fatty acyl composition corresponding to PG 17:3_18:2 + PG 17:2_18:3 and PG 15:0_20:5, respectively.


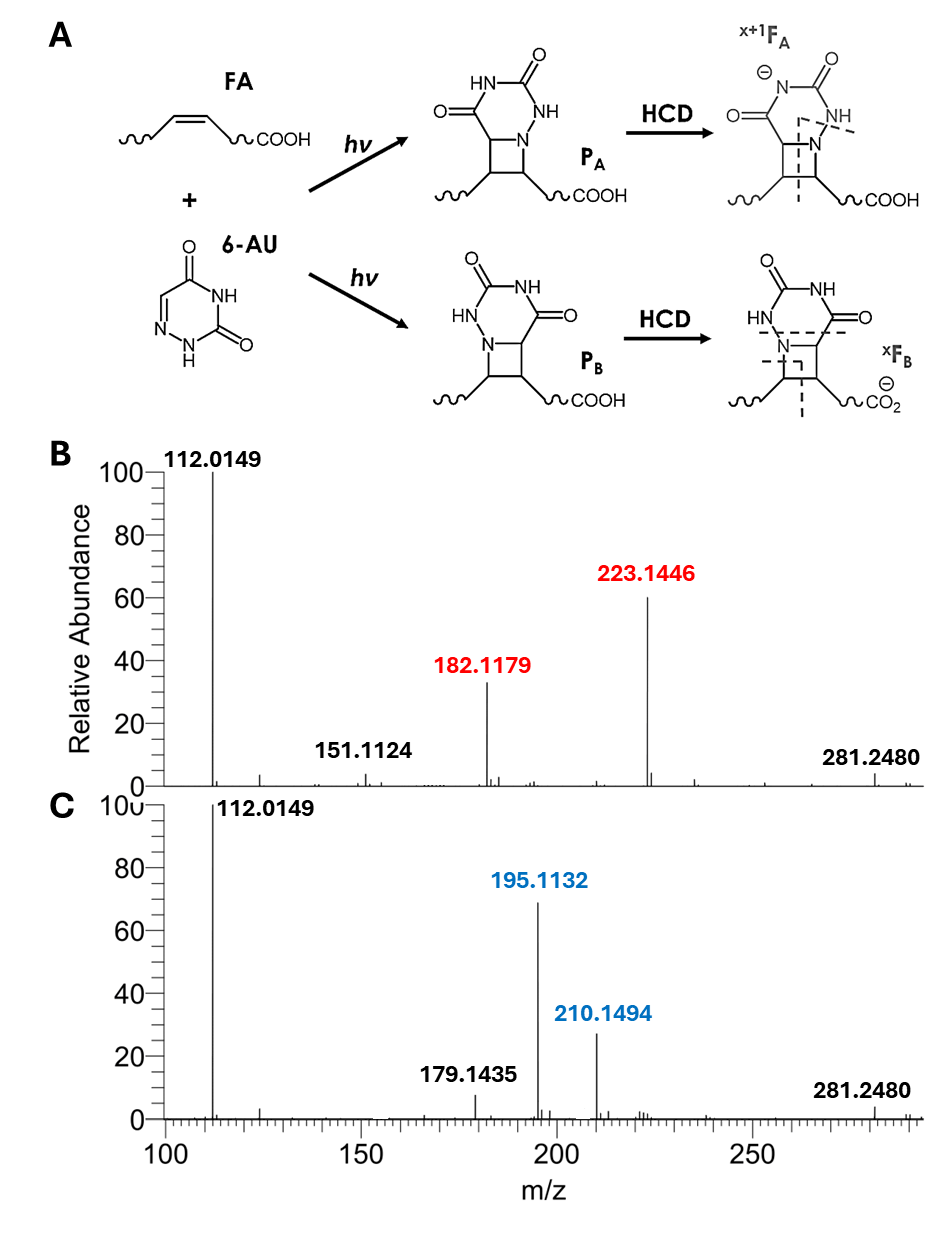


**Figure S14.** Scheme of the aPB reaction of pinpointing carbon-carbon double bonds on fatty acyl chains (A). MS/MS spectra (30 NCE) of the aPB derivatives of FA 18:1 ω -9 (B) and ω -7 (C). Diagnostic product ions are marked in red and blue for n-9 and n-7 respectively.


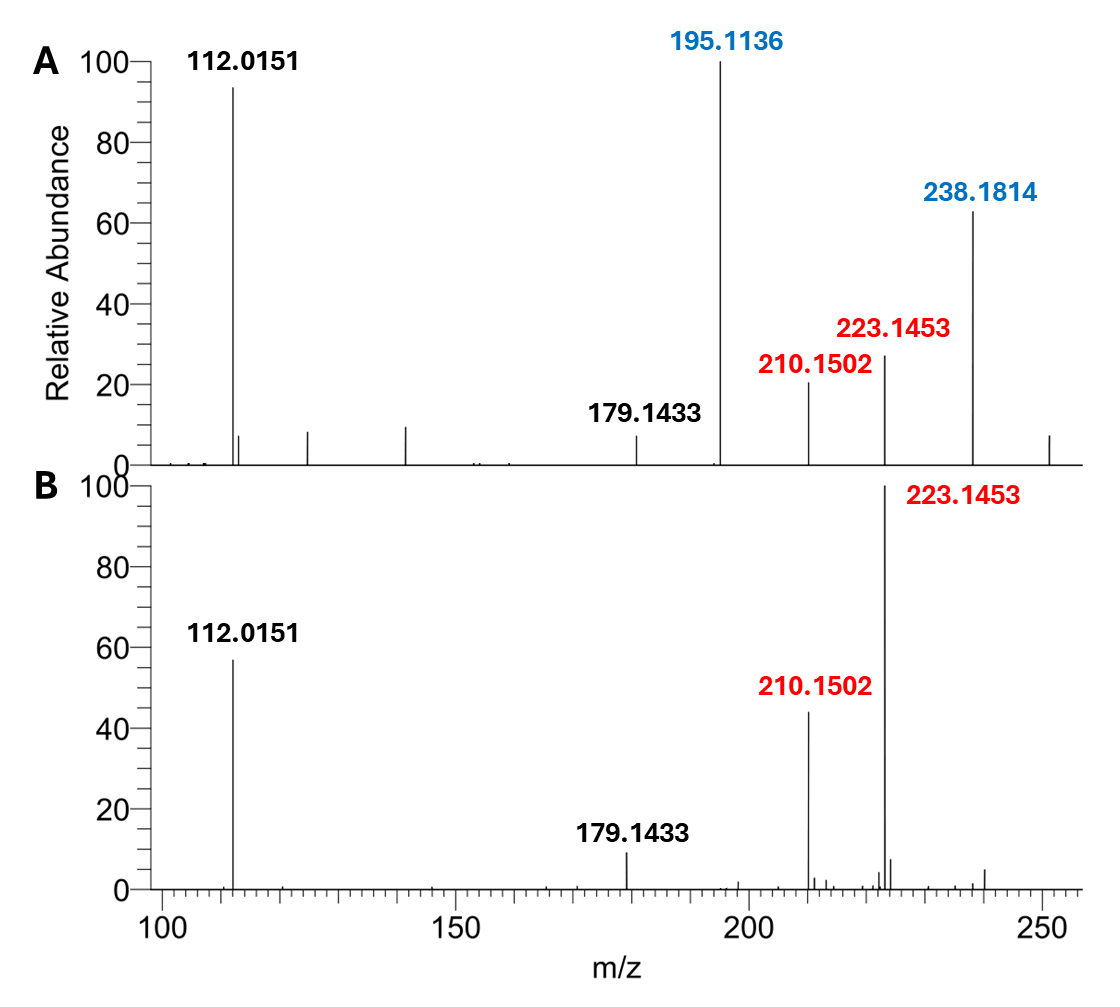


**Figure S15.** MS/MS spectra (30 NCE) of the aPB derivatives of FA 20:1 from Carraghen (A) and Nori (B). Diagnostic product ions are marked in red and blue for ω-9 and ω-7, respectively.


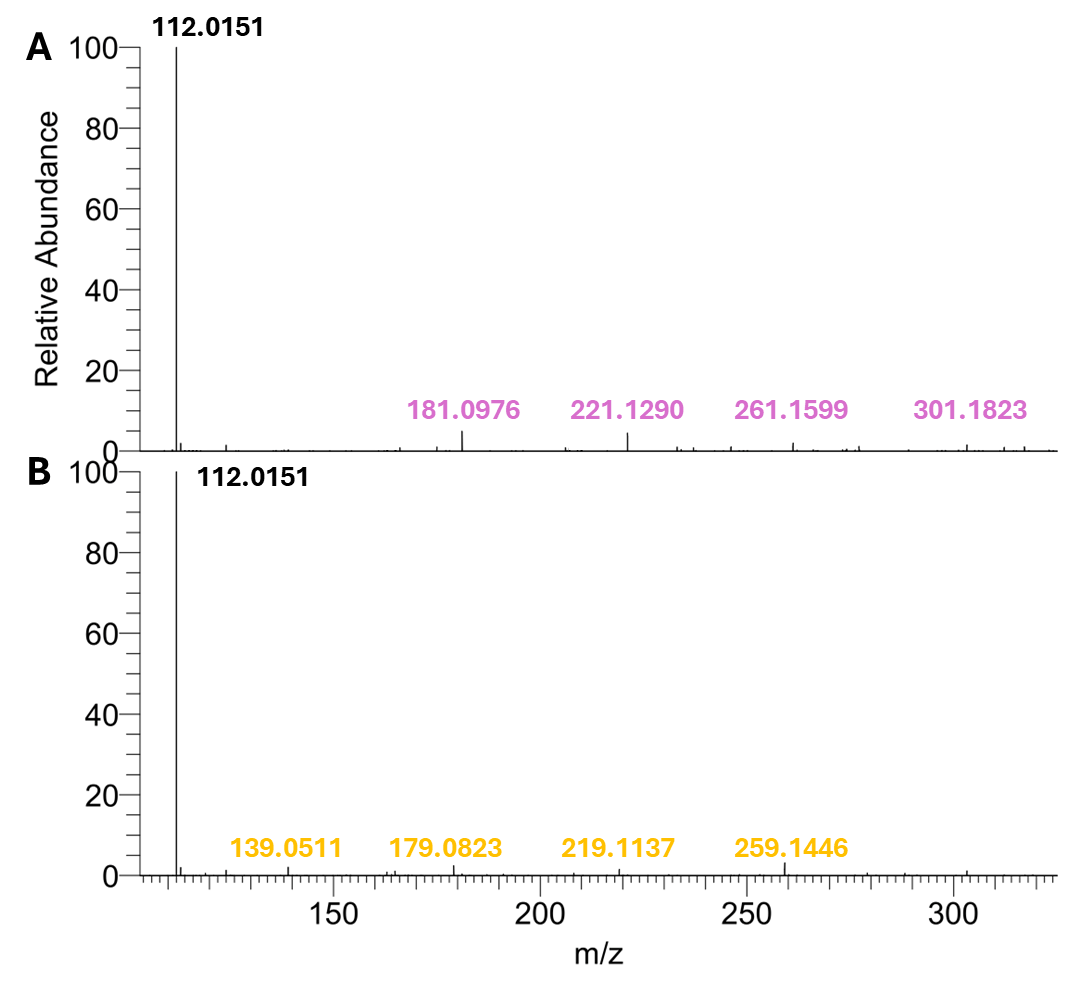


**Figure S16.** MS/MS spectra (30 NCE) of the aPB derivatives of FA 20:4 from Dulse (A) and Sea Lettuce (B). Diagnostic product ions are marked in purple and yellow for ω-6 and ω-3, respectively.
